# Pooled Resistance Analysis in Patients with Hepatitis C Virus Genotype 1 to 6 Infection Treated with Glecaprevir-Pibrentasvir in Phase 2 and 3 Clinical Trials

**DOI:** 10.1128/AAC.01249-18

**Published:** 2018-09-24

**Authors:** Preethi Krishnan, Tami Pilot-Matias, Gretja Schnell, Rakesh Tripathi, Teresa I. Ng, Thomas Reisch, Jill Beyer, Tatyana Dekhtyar, Michelle Irvin, Wangang Xie, Lois Larsen, Federico J. Mensa, Christine Collins

**Affiliations:** a Research & Development, AbbVie, Inc., North Chicago, Illinois, USA

**Keywords:** HCV genotype, NS5A inhibitor, direct acting antiviral, glecaprevir, hepatitis C virus, pibrentasvir, protease inhibitor

## Abstract

Over 2,200 patients infected with hepatitis C virus (HCV) genotypes (GT) 1 to 6, with or without cirrhosis, who were treatment naive or experienced to interferon, ribavirin, and/or sofosbuvir were treated with glecaprevir/pibrentasvir for 8, 12, or 16 weeks in eight registrational phase 2 and 3 clinical studies. High rates of sustained virologic response at 12 weeks postdosing (SVR12) were achieved with a <1% virologic failure (VF) rate. The prevalence of baseline polymorphisms (BPs) in NS3 at amino acid position 155 or 168 was low (<3%) in patients infected with GT1, GT2, GT3, GT4, and GT6, while 41.9% of the GT5-infected patients had NS3-D168E; BPs were not detected at position 156 in NS3. The prevalence of NS5A-BPs was high across genotypes, driven by common polymorphisms at amino acid position 30 or 31 in GT2, 58 in GT4, and 28 in GT6. The prevalence of NS5A T/Y93 polymorphisms was 5.5% in GT1, 4.9% in GT3, and 12.5% in GT6. Consistent with the activity of glecaprevir and pibrentasvir against most amino acid polymorphisms *in vitro*, BPs in NS3 and/or NS5A did not have an impact on treatment outcome for patients infected with GT1 to GT6, with the exception of treatment-experienced GT3-infected patients treated for 12 weeks, for whom a 16-week regimen of glecaprevir/pibrentasvir was required to achieve SVR12 rates of ≥95%. Among the 22 patients experiencing VF, treatment-emergent substitutions were detected in NS3 in 50% of patients and in NS5A in 82% of patients, frequently as a combination of substitutions that conferred resistance to glecaprevir and/or pibrentasvir. The glecaprevir/pibrentasvir regimen, when the recommended durations are used, allows for a pan-genotypic treatment option without the need for baseline resistance testing.

## Abstract (precis)

Over 2,200 patients infected with hepatitis C virus (HCV) genotypes (GT) 1 to 6, with or without cirrhosis, who were treatment naive or experienced to interferon, ribavirin, and/or sofosbuvir were treated with glecaprevir/pibrentasvir for 8, 12, or 16 weeks in eight registrational phase 2 and 3 clinical studies. High rates of sustained virologic response at 12 weeks postdosing (SVR12) were achieved with a <1% virologic failure (VF) rate.

## INTRODUCTION

Hepatitis C virus (HCV) infection is a global health problem, with over 70 million individuals chronically infected worldwide (1). There are seven HCV genotypes (GTs) and 67 subtypes, with genotype distribution varying by geographic locations (2). The majority of infections in North America, South America, and Europe are with HCV GT1. HCV GT2 and GT3 infections are common in Latin America (5% to 30%), Europe (20% to 40%), and Asia (30% to 45%) (3–5). HCV GT4 is commonly found in parts of Africa and the Middle East, particularly in Egypt, GT5 is primarily found in South Africa, and GT6 is primarily found in Southeast Asia (3–5). To date, only a few GT7 isolates have been reported, all of which were found in patients who originated from the Democratic Republic of Congo (2, 6–8).

The level of HCV nucleotide sequence diversity ranges from 30 to 35% between genotypes and from 20 to 25% between subtypes. HCV has a high rate of replication, and it is estimated that 10^12^ virions are produced per day in an infected individual (9). The RNA-dependent RNA polymerase of HCV is intrinsically error prone, and its lack of a proofreading function results in the presence of preexisting drug-resistant variants in infected patients and the expansion of these resistant species under the selective pressure of an HCV inhibitor (10). Though therapy for HCV infection has improved considerably with the availability of several interferon (IFN)-free direct-acting antiviral (DAA) regimens, most of the approved and recommended regimens, including ombitasvir/paritaprevir/ritonavir with or without dasabuvir, grazoprevir/elbasvir, ledipasvir/sofosbuvir, sofosbuvir/velpatasvir, or sofosbuvir/velpatasvir/voxilaprevir, are not equally effective across all HCV genotypes and subpopulations. Baseline amino acid polymorphisms associated with reduced susceptibility to HCV nonstructural (NS) viral protein NS3/4A protease inhibitors (especially Q80K) or NS5A inhibitors (especially Y93H) are associated with reduced treatment efficacy for several DAA regimens in some HCV subtypes or subpopulations, requiring longer treatment durations or addition of ribavirin (RBV) (11–14). Efficacy of approved DAA regimens in GT3-infected patients, particularly in those who are treatment experienced and/or cirrhotic, is also less than optimal and substantially lower than that observed for other genotypes (15–17).

Glecaprevir, an HCV NS3/4A protease inhibitor (identified by AbbVie and Enanta), and pibrentasvir, an NS5A inhibitor, are next-generation HCV inhibitors with *in vitro* antiviral activity against genotypes 1 through 6, with no or little loss of potency against common resistance-associated amino acid substitutions (18, 19). Glecaprevir at 300 mg and pibrentasvir at 120 mg (without RBV), coformulated into a fixed-dose combination tablet taken once daily with food, were evaluated as a pan-genotypic regimen in eight phase 2 and 3 clinical studies: SURVEYOR-1 and -2, ENDURANCE-1, -2, -3, and -4, and EXPEDITION-1 and -4 (20–28). The trials evaluated glecaprevir/pibrentasvir for 8, 12, and 16 weeks in patients chronically infected with GT1, -2, -3, -4, -5 and -6 and compensated liver disease (with and without cirrhosis), including treatment-naive (TN) patients and treatment-experienced (TE) patients treated with pegylated IFN (peg-IFN) and RBV with or without sofosbuvir (TE-PRS), patients with HIV coinfection, and patients with advanced renal disease. The rate for the pooled overall sustained virologic response at 12 weeks postdosing (SVR12) was 98% in 2,256 patients, with a <1% virologic failure rate (22 of 2,256 patients) (29).

The pooled resistance analysis of the eight phase 2 and 3 studies is presented in this report, including HCV subtype distribution, prevalence of baseline polymorphisms and their impact on treatment outcome in patients grouped by HCV subtype, duration of treatment with glecaprevir/pibrentasvir, prior treatment history (TN or TE-PRS), and cirrhosis status. Treatment-emergent substitutions in the patients experiencing virologic failure in the phase 2 and 3 studies are also characterized.

## RESULTS

### HCV subtype distribution in phase 2 and 3 clinical studies.

**FIG 1:**
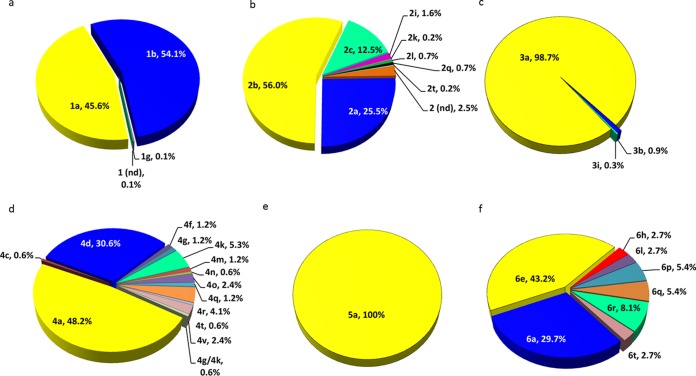
HCV subtype distribution in phase 2 and 3 studies with glecaprevir/pibrentasvir. HCV subtypes were assigned based on the phylogenetic analysis of available full-length HCV NS3/4A and/or NS5A consensus nucleotide sequences as follows: GT1, *n* = 861 (a); GT2, *n* = 439 (b); GT3, *n* = 635 (c); GT4, *n* = 170 (d); GT5, *n* = 31 (e); GT6, *n* = 37 (f). nd, the sequence did not align with any known subtype within the respective genotype.

Phylogenetic analysis of HCV from 2,173 patients with available baseline NS3/4A and/or NS5A sequences out of the 2,256 patients in the intent-to-treat (ITT) population (defined as patients who took at least one dose of glecaprevir/pibrentasvir) identified 38 HCV subtypes in the SURVEYOR-1 and -2, ENDURANCE-1, -2, -3, and -4, and EXPEDITION-1 and -4 studies. This included 3 GT1 (*n* = 861, predominantly GT1a and GT1b), 8 GT2 (*n* = 439, predominantly GT2a, GT2b, and GT2c), 3 GT3 (*n* = 635, predominantly GT3a), 14 GT4 (*n* = 170, predominantly GT4a and GT4d), 1 GT5 (*n* = 31, GT5a), and 8 GT6 (*n* = 37, predominantly GT6a and GT6e) subtypes (Fig. 1). HCV genotype and subtype were determined by Inno-LiPA (line probe assay), version 2.0, or Sanger sequencing of a small region of the NS5B gene before study enrollment and were subsequently compared to the assessment by phylogenetic analysis of available NS3/4A and/or NS5A sequences (data not shown). Genotype assignment by LiPA generally matched genotype assignment by phylogenetic analyses except for 11 patients assigned as GT2 by LiPA who were determined to be GT1 by phylogenetic analysis of NS3/4A and NS5A sequences and were considered GT1 in resistance analyses. HCV genotype was assigned based on the results of the LiPA or the Sanger sequencing assay for 83 patients without available NS3/4A or NS5A sequence data.

### Efficacy of glecaprevir/pibrentasvir in a pooled analysis of phase 2 and 3 studies.

Among the 2,256 patients in the ITT population in eight phase 2 and 3 registrational studies, 5 patients experienced on-treatment virologic failure, 17 patients experienced relapse, 1 patient experienced HCV reinfection, 12 patients prematurely discontinued study drugs, and 15 patients were missing viral load data for the SVR12 time point. The SVR12 rates in the modified-ITT patient population (excluding the 28 patients not achieving SVR12 due to nonvirologic reasons for failure such as premature study drug discontinuation, missing SVR12 HCV RNA value, or reinfection) were grouped by HCV genotype, treatment duration, treatment experience, and cirrhosis status (Table 1). Twenty-two patients experienced virologic failure, including 2 patients with GT1a, 2 with GT2a, 17 with GT3a, and 1 with GT3b infection.

**TABLE 1:** SVR12 in pooled analysis of phase 2 and 3 registrational studies with glecaprevir/pibrentasvir in the modified-ITT population^*a*^

HCV GT	SVR12 rate (%) by patient population, prior treatment experience, and treatment duration^*b*^							
	No cirrhosis					Compensated cirrhosis		
	TN (8 wk)	TE-PRS (8 wk)	TN (12 wk)	TE-PRS (12 wk)	TE-PRS (16 wk)	TN (12 wk)	TE-PRS (12 wk)	TE-PRS (16 wk)
1	100 (245/245)	99 (138/139)	100 (241/241)	100 (159/159)	—	100 (69/69)	97 (29/30)	—
2	100 (172/172)	91 (21/23)	99 (167/167)	100 (65/65)	—	100 (26/26)	100 (9/9)	—
3	95 (177/183)	—	96 (258/261)	90 (44/49)	96 (21/22)	100 (64/64)	—	94 (48/51)
4	100 (37/37)	100 (7/7)	100 (71/71)	100 (40/40)	—	100 (12/12)	100 (8/8)	—
5	100 (2/2)		100 (22/22)	100 (6/6)	—	100 (2/2)		—
6	100 (8/8)	100 (2/2)	100 (27/27)	100 (4/4)	—	100 (6/6)	100 (1/1)	—

a Patients not achieving SVR12 for nonvirologic reasons such as premature study drugs discontinuation, missing SVR12 data, or reinfection were excluded in the modified-intent-to-treat analysis.

b Values in parentheses are the number of patients who achieved SVR12/total number of patients in the group. TE-PRS, treatment-experienced to peg-IFN plus RBV with or without sofosbuvir; TN, treatment-naive; —, patients with this HCV genotype were excluded from treatment for that duration, per enrollment criteria.

### Prevalence of baseline polymorphisms in patients infected with GT1, GT2, GT4, GT5, and GT6.

**FIG 2:**
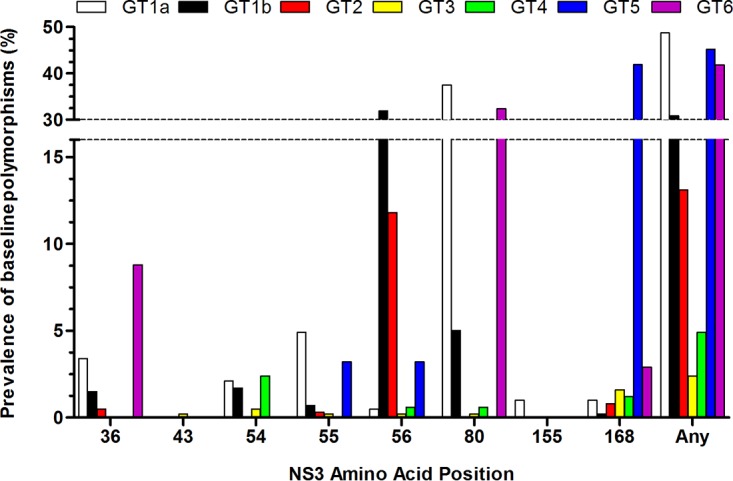
Prevalence of baseline polymorphisms in NS3. The figure shows the percentage of patients with baseline polymorphisms at amino acid positions of interest for NS3/4A protease inhibitors relative to a subtype-specific reference sequence at a 15% detection threshold. For GT2 to GT6, analysis combined polymorphisms at each amino acid position across subtypes. Baseline polymorphisms were not detected at amino acid position 156 in any genotype. GT1a, *n* = 384; GT1b, *n* = 461; GT2, *n* = 398; GT3, *n* = 613; GT4, *n* = 164; GT5, *n* = 31; GT6, *n* = 34.

**FIG 3:**
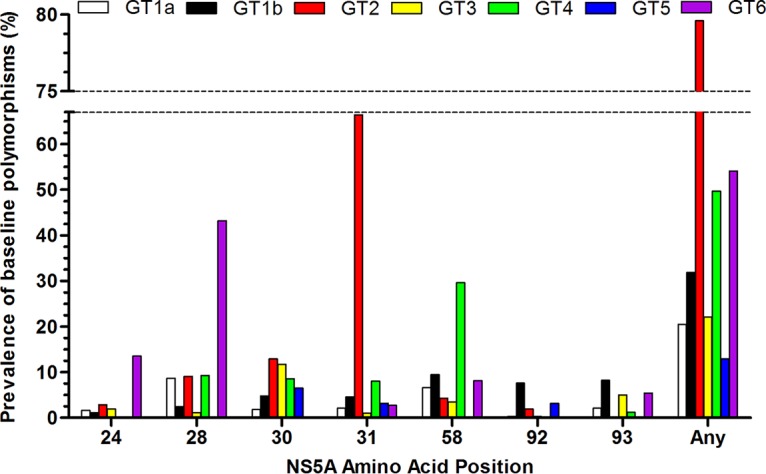
Prevalence of baseline polymorphisms in NS5A. The figure shows the percentage of patients with baseline polymorphisms at amino acid positions of interest for NS5A inhibitors relative to the appropriate subtype-specific reference sequence at a 15% detection threshold. For GT2 to GT6, analysis combined polymorphisms at each amino acid position across subtypes. Baseline polymorphisms were not detected at amino acid position 32 in any genotype. GT1a, *n* = 380; GT1b, *n* = 461; GT2, *n* = 415; GT3, *n* = 615; GT4, *n* = 161; GT5, *n* = 31; GT6, *n* = 37.

Baseline polymorphisms relative to the appropriate subtype-specific reference sequences were analyzed at amino acid positions at which substitutions have been observed *in vitro* or clinically in NS3 or NS5A with drugs for the respective inhibitor class. These included amino acid positions 36, 43, 54, 55, 56, 80, 155, 156, and 168 in NS3 and positions 24, 28, 30, 31, 32, 58, 92, and 93 in NS5A (Fig. 2 and Fig. 3). The most commonly occurring amino acid at each of these positions in each subtype-specific reference sequence is shown in Tables S1 and S2 in the supplemental material. The prevalence of each baseline polymorphism at these amino acid positions relative to the appropriate subtype-specific reference sequence in the patients in the eight clinical studies is detailed in Table S3.

Individual amino acid substitutions associated with resistance to other HCV NS3/4A protease inhibitors at amino acid position 36, 43, 54, 55, 56, 155, 166, or 170 in NS3 generally did not confer resistance to glecaprevir (19). Substitutions at NS3 amino acid position A156 conferred the greatest reductions (>100-fold) in susceptibility to glecaprevir in most genotypes, while those at position (D/Q)168 had various effects on glecaprevir susceptibility, depending on HCV subtype and specific amino acid changes. Baseline NS3 polymorphisms at amino acid position 155 or 168 were rare in GT1, -2, -3, -4, and -6, while D168E in GT5 was detected in 41.9% (13/31) of the patients. Baseline polymorphisms at amino acid position 156 were not detected in any HCV genotype (Fig. 2). Q80(K/L/M/R) polymorphisms were detected in 37.5% (144/384) of GT1a-infected patients, with Q80K, which does not confer resistance to glecaprevir, making up the majority of these; L80K was detected in 100% (11/11) of the GT6a-infected patients (Table S3).

The majority of individual amino acid substitutions associated with resistance to other HCV NS5A inhibitors at amino acid position 24, 28, 30, 31, 58, 92, or 93 in NS5A did not confer resistance to pibrentasvir (18). Prevalence of baseline NS5A polymorphisms was 12.9% in GT1-infected and 26.8% in GT5-infected patients and ranged between 49.7% and 79.8% in GT2-, GT4-, and GT6-infected patients (Fig. 3). The high prevalence of baseline NS5A polymorphisms was driven by the common (L/M)31 polymorphism in GT2a and GT2b and R30K in GT2c, polymorphisms at amino acid position 58 in GT4a, GT4d and GT4f, and polymorphisms at amino acid position 28 in GT6 (Table S3). The prevalence of Y93(C/F/H/N/S) polymorphisms in GT1a- and GT1b-infected patients was 2.1% (8/380) and 8.2% (38/461), respectively, and T93S was detected in 12.5% (2/16) of the GT6e-infected patients (Table S3). NS5A-Y93 substitutions in subgenomic replicons across genotypes remained susceptible to pibrentasvir (14, 18).

Within each genotype and drug target, the prevalences of baseline polymorphisms in patients were similar across treatment durations, prior treatment experience, or cirrhosis status (data not shown).

### Baseline polymorphisms and treatment-emergent substitutions in GT1a- and GT2a-infected patients experiencing virologic failure.

Among GT1a-infected patients, 1 of 38 TE-PRS patients without cirrhosis who received 8 weeks of treatment and 1 of 12 patients with cirrhosis who received 12 weeks of treatment experienced virologic failure (Table 2). Neither patient had baseline polymorphisms in NS3; one patient had Y93N in NS5A. Among GT1a-infected patients grouped by treatment duration, prior treatment experience, and cirrhosis status, baseline polymorphisms in NS3 and NS5A, including NS3-Q80K and NS5A-Y93 polymorphisms, had no impact on SVR12 rates (Table 3).

**TABLE 2:** Baseline polymorphisms and treatment-emergent substitutions in four GT1- and GT2-infected patients experiencing virologic failure

HCV GT and patient no.^*a*^	Duration (wks)	Cirrhosis (Y/N)^*b*^	Outcome^*c*^	Variant(s) by time point (prevalence [%] within patient's viral population)^*d*^					
				NS3			NS5A		
				Baseline	Time of VF	Posttreatment wk 24	Baseline	Time of VF	Posttreatment wk 24
1a									
1	8	N	BT	None	A156V (99.9)	NA	None	Q30R (98.5), L31M (99.6), H58D (97.4)	NA
2	12	Y	R	None	None	None	Y93N (46.2)	Q30R (87.8), Y93N (99.7)	Q30R (81.8), H58D (21.1), Y93N (99.8)
2a									
1	8	N	R	None	None	None	L31M (99.4)	L31M (99.9)	L31M (99.8)
2	8	N	R	None	None	None	L31M (99.7)	L31M (99.9)	L31M (99.9)

a All four patients belonged to the TE-PRS category (prior experience to peg-IFN plus RBV with or without sofosbuvir).

b Y, yes; N, no.

c BT, breakthrough; R, relapse.

d Variants at signature amino acid positions relative to subtype-specific reference sequences in NS3 and NS5A at a 15% detection threshold are listed. None, polymorphisms or substitutions at signature amino acid positions were not detected; NA, not available; VF, virologic failure.

**TABLE 3:** SVR12 rates in the presence and absence of baseline polymorphisms in NS3 or NS5A in GT1a-infected patients

Target and polymorphism^*a*^	SVR12 rate (%) by patient population, prior treatment experience, and treatment duration^*b*^					
	No cirrhosis				Compensated cirrhosis	
	TN (8 wk)	TE-PRS (8 wk)	TN (12 wk)	TE-PRS (12 wk)	TN (12 wk)	TE-PRS (12 wk)
NS3						
Polymorphism at aa 155, 156, or 168	100 (5/5)		100 (1/1)	100 (1/1)	100 (1/1)	
No polymorphisms	100 (129/129)	97.3 (36/37)	100 (112/112)	100 (47/47)	100 (39/39)	91.7 (11/12)
With Q80K	100 (48/48)	100 (12/12)	100 (40/40)	100 (9/9)	100 (21/21)	100 (5/5)
Without Q80K	100 (86/86)	96.0 (24/25)	100 (73/73)	100 (39/39)	100 (19/19)	85.7 (6/7)
NS5A						
Polymorphism at aa 24, 28, 30, 31, 58, 92, or 93	100 (27/27)	100 (9/9)	100 (24/24)	100 (6/6)	100 (10/10)	50.0 (1/2)^*c*^
No polymorphisms	100 (105/105)	96.6 (28/29)	100 (86/86)	100 (41/41)	100 (29/29)	100 (12/12)

a Polymorphisms relative to subtype-specific reference sequence at a 15% detection threshold. aa, amino acid.

b Values in parentheses are the number of patients who achieved SVR12/total number of patients in the group with available sequence. TE-PRS, treatment-experienced to peg-IFN plus RBV with or without sofosbuvir; TN, treatment-naive.

c Patient experiencing virologic failure had a Y93N substitution in NS5A at baseline.

One of the two GT1a-infected patients experiencing virologic failure had the treatment-emergent substitution A156V in NS3; however, a posttreatment week 24 sample from this patient was not available for analysis, so persistence of this substitution could not be assessed. Long-term follow-up studies evaluating persistence of NS3-A156 substitutions in patients experiencing virologic failure with telaprevir-containing regimens have shown that median time to loss of A156 substitutions ranges from 1.8 to 8.2 months (30). In the GT1a replicon, the NS3-A156V substitution resulted in poor replication capacity, and the half-maximal effective concentration (EC_50_) of glecaprevir could not be evaluated (Table 4). Both GT1a-infected patients experiencing virologic failure had treatment-emergent substitutions in NS5A. Amino acid substitutions Q30R, L31M, and H58D were detected in one patient at the time of failure, but the posttreatment week 24 sample was not available for analysis. The other patient had a Q30R substitution in addition to the preexisting Y93N at the time of failure, and Q30R, Y93N, and an additional substitution, H58D, were detectable at posttreatment week 24 (Table 2). In the GT1a replicon, single substitutions Q30R, L31M, or H58D remained susceptible to pibrentasvir, while the Y93N substitution conferred 7-fold resistance. The combinations detected in the two patients at the time of failure conferred 23- to 1,704-fold resistance to pibrentasvir (Table 4).

**TABLE 4:** *In vitro* activity of glecaprevir or pibrentasvir against amino acid substitutions in GT1a and GT3a replicons^*a*^

HCV GT and variant	Mean glecaprevir EC_50_ ± SD (nM)	Mean pibrentasvir EC_50_ ± SD (pM)	Fold change in EC_50_
1a			
NS3			
Wild type	0.21 ± 0.08		
Q80K	0.19 ± 0.05		0.9
A156V	NA		
NS5A			
Wild type		0.72 ± 0.45	
Q30R		1.2 ± 0.62	1.7
L31M		0.76 ± 0.11	1.1
H58D		0.80 ± 0.17	1.1
Y93N		5.1 ± 2.1	7.0
Q30R + L31M		2.1 ± 0.79	3.0
Q30R + Y93N		94.6 ± 15.5	131
L31M + H58D		16.6 ± 2.7	23
Q30R + L31M + H58D		1,227 ± 277	1,704
3a			
NS3			
Wild type^*b*^	0.55 ± 0.17		
Y56H	NA		
Q80R	11.5 ± 1.6		21
A156G	909 ± 349		1654
S166A	NA		
S166T	2.6 ± 0.58		4.7
Q168L	6.9 ± 1.7		13
Q168R	30.0 ± 10.4		54
Y56H + S166A	NA		
Y56H + Q168L	NA		
Y56H + Q168R	763 ± 363		1387
S166A + Q168(K/L/R)	NA		
NS5A			
Wild type		0.65 ± 0.16	
M28G		NA	
A30K		0.71 ± 0.18	1.1
L31F		NA	
Y93H		1.5 ± 0.19	2.3
A30K + Y93H		45.1 ± 2.3	69
L31F + Y93H		NA	

a EC_50_, 50% effective concentration. Fold change was determined relative to the wild-type value. NA, not available due to low replication efficiency of the replicon containing the amino acid substitution; SD, standard deviation.

b The wild-type replicon has S166 in NS3.

Two of the 31 (including 24 TN and 7 TE-PRS) GT2a-infected patients without cirrhosis, who had received treatment for 8 weeks, experienced virologic failure (Table 2). Neither patient had baseline polymorphisms in NS3 or NS5A, except for the common NS5A-M31 polymorphism. Treatment-emergent substitutions were not detected in NS3 or NS5A in either patient (Table 2). Lack of baseline polymorphisms or treatment-emergent substitutions suggested that both patients had experienced virologic failure for reasons unrelated to drug resistance.

No patient infected with GT1b, GT1g, GT2 (non-2a), GT4, GT5, or GT6 experienced virologic failure (Table 1). Baseline polymorphisms in NS3 or NS5A had no impact on treatment outcome in patients infected with any GT1, GT2, GT4, GT5, or GT6 subtype, irrespective of treatment duration, prior treatment experience, or cirrhosis status, consistent with the observation that glecaprevir and pibrentasvir retained their *in vitro* activity against most amino acid substitutions in NS3 and NS5A, respectively, for these genotypes (18, 19).

### Prevalence of baseline polymorphisms in GT3-infected patients.

The majority of GT3-infected patients enrolled in the phase 2 and 3 clinical studies were infected with subtype 3a (627 of 635 based on phylogenetic analysis) (Fig. 1). Baseline polymorphisms at amino acid position 36, 43, 54, 55, 56, or 80 in NS3 were each detected in <1% of the patients (Table 5). NS3 A166(S/T) polymorphisms were detected in 13.9% of the GT3a-infected patients (84/605; one patient had a mixture of A166S and A166T). The laboratory strain wild-type GT3a chimeric replicon used to evaluate the activity of glecaprevir *in vitro* had S166 in NS3, and the EC_50_ of glecaprevir against the GT3a wild-type replicon (0.55 nM) was comparable to the EC_50_ against the GT1a wild-type replicon (0.21 nM) (Table 4). A replicon with NS3-A166 had poor replication capacity and could not be evaluated, and the EC_50_ of glecaprevir against a replicon with NS3-T166 was 2.6 nM (Table 4). Q168(K/R) polymorphisms in NS3 were detected in 1.7% (10/605) of the GT3a-infected patients at baseline; a replicon with NS3-Q168K had poor replication capacity, and a Q168R substitution conferred 54-fold resistance to glecaprevir (Table 4). Baseline NS3 polymorphisms were not detected in the 6 GT3b- or 2 GT3i-infected patients.

**TABLE 5:** Prevalence of baseline polymorphisms in NS3 and/or NS5A in GT3-infected patients

HCV GT	NS3 polymorphism^*a*^		NS5A polymorphism^*a*^	
	Type^*b*^	Prevalence (%)^*c*^	Type	Prevalence (%)
3a	Any NS3	15.9 (96/605)	Any NS5A	21.4 (130/607)
	F43L	0.2 (1/605)	S24A	2.0 (12/607)
	T54(A/S)	0.5 (3/605)	M28V	1.2 (7/607)
	V55I	0.2 (1/605)	A30(L/M/R/S/T/V)	5.9 (36/607)
	Y56F	0.2 (1/605)	A30K	6.4 (39/607)
	Q80K	0.2 (1/605)	P58(A/R/S/T/Y)	3.6 (22/607)
	A166S	9.1 (55/605)	E92(D/G)	0.3 (2/607)
	A166T	5.0 (30/605)	Y93H/F	4.9 (30/607)
	Q168K	1.0 (6/605)		
	Q168R	0.7 (4/605)		
3b	Any NS3	(0/6)	Any NS5A	100 (6/6)
			K30M	16.7 (1/6)
			V31M	100 (6/6)
3i	Any NS3	(0/2)	Any NS5A	(0/2)
All GT3	Any NS3	15.7 (96/613)	Any NS5A	22.1 (136/615)

a Polymorphisms relative to subtype-specific reference sequences at a 15% detection threshold at amino acid position 36, 43, 54, 55, 56, 80, 155, 156, 166 or 168 in NS3 or position 24, 28, 30, 31, 32, 58, 92, or 93 in NS5A.

b “Any” indicates the total number of patients with any polymorphism within each target.

c Values in parentheses are the number of patients with the polymorphism/total number of patients in the group with available sequence.

Baseline polymorphisms at amino acid position 24, 28, or 92 in NS5A were rare; and those at position 58 were detected in 3.6% (22/607) of the GT3a-infected patients (Table 5). The prevalence of NS5A-A30 polymorphisms was 11.7% (71/607), of which the A30K substitution was detected in 6.4% (39/607) of the GT3a-infected patients. NS5A-Y93H was detected in 4.9% (30/607; one patient had a mixture of Y93F and Y93H). In the GT3a NS5A chimeric replicon, the A30K or Y93H substitution each conferred <3-fold resistance to pibrentasvir (Table 4). In GT3b, the NS5A reference sequence has K30 and V31 (Table S2); 5 of 6 GT3b-infected patients had K30 in NS5A, while 1 patient had a K30M substitution. All 6 GT3b-infected patients had V31M (Table 5). Pibrentasvir had an EC_50_ of 15.6 pM against a GT3b NS5A chimeric replicon containing K30 and M31 in NS5A as compared to an EC_50_ of 0.65 pM against a GT3a NS5A chimeric replicon (Table 6). Baseline NS5A polymorphisms were not detected in the 2 GT3i-infected patients.

**TABLE 6:** *In vitro* activity of NS5A inhibitors against chimeric NS5A GT3b HCV replicons

HCV replicon	Amino acid at positions of interest in NS5A	Mean EC_50_ ± SD (pM)^*e*^		
		Pibrentasvir	Daclatasvir	Velpatasvir
GT3a^*a*^				
Wild type	S24, M28, A30, L31, P32, P58, E92, Y93	0.65 ± 0.16	23.3 ± 7.9	4.4 ± 0.89
Wild type (JFH1)		0.39 ± 0.08	61.4 ± 22.5	1.84 ± 0.31
GT3b^*b*^				
Wild-type	S24, M28, K30, M31, P32, P58, E92, Y93	15.6 ± 1.5	1,267,333 ± 74,097	200,567 ± 41,464
K30A^*c*^	S24, M28, A30, M31, P32, P58, E92, Y93	0.62 ± 0.04	35,263 ± 12,276	195 ± 43
M31L^*c*^	S24, M28, K30, L31, P32, P58, E92, Y93	2.5 ± 0.5	579,300 ± 127,525	10,188 ± 1,513
K30A + M31L^*c*^	S24, M28, A30, L31, P32, P58, E92, Y93	0.91 ± 0.12	169 ± 19.3	4.97 ± 1.37
M31V^*d*^	S24, M28, K30, V31, P32, P58, E92, Y93	365 ± 47	1,690,667 ± 268,658	346,867 ± 119,158
Y93H	S24, M28, K30, M31, P32, P58, E92, H93	98,843 ± 35,901	2,795,333 ± 677,856	1,607,000 ± 659,190

a GT3a wild type refers to NS5A-GT3a chimeric replicon in GT1b-Con1 background. This replicon was used to evaluate activity of pibrentasvir as shown in Table 4. The JFH1 wild-type refers to NS5A-GT3a chimeric replicon in a GT2a-JFH1 background.

b GT3b wild-type refers to NS5A-GT3b chimeric replicon in the GT2a-JFH1 background; GT3b amino acid substitutions were also generated in this chimeric replicon.

c The polymorphism was not detected in patient isolates. Substitutions K30A and/or M31L were constructed to evaluate their potential impact on susceptibility to NS5A inhibitors.

d The polymorphism was not detected in patient isolates. Substitution M31V was constructed to match amino acids at positions of interest to those in reference sequence HCV-Tr.

e EC_50_, half-maximal effective concentration; SD, standard deviation.

### Baseline polymorphisms and treatment-emergent substitutions in GT3-infected patients experiencing virologic failure.

In the pooled analysis, 17 patients with GT3a and 1 patient with GT3b infection experienced virologic failure (Table 7). Three of the 18 patients experiencing virologic failure had not adhered to the treatment regimen according to pill counts (see Materials and Methods) but were included in the modified-ITT population.

**TABLE 7:** Baseline polymorphisms and treatment-emergent substitutions in 18 GT3-infected patients experiencing virologic failure

Prior treatment experience and duration^*a*^	Patient no.	Cirrhosis (Y/N)^*d*^	Outcome^*e*^	Variant(s) by time point (prevalence [%] within patient's viral population)^*g*^					
				NS3			NS5A		
				Baseline	Time of VF^*f*^	Posttreatment wk 24	Baseline	Time of VF	Posttreatment wk 24
TN									
8 wk	1^*b*^	N	BT	A166S (60.6), Q168R (61.4)	Q80R (59.2), A156G (99.6)	A166S (85.4), Q168R (85.6)	A30K (99.6)	A30K (99.9), Y93H (99.7)	A30K (99.8), Y93H (99.1)
	2	N	R	A166S (52.4)	Y56H (88.5), Q168L (95.5)	Q168L (99.3)	A30K (99.8)	A30K (99.8), Y93H (99.8)	A30K (99.8), Y93H (99.6)
	3	N	R	T54S (98.4)	T54S (99.4)	T54S (99.7)	None	None	None
	4	N	R	A166S (99.2)	A166S (97.7)	A166S (99.9)	None	Y93H (99.6)	Y93H (99.6)
	5	N	R	None	Y56H (99.5)	None	A30K (99.8)	A30K (99.8), Y93H (99.6)	A30K (99.9)
	6	N	R	None	Q168L (98.9)	None	A30K (99.7)	A30K (99.8), Y93H (99.7)	A30K (99.9), Y93H (77.1)
12 wk	7	N	BT	Q168R (28.5)	Y56H (98.5), Q168R (99.2)	None	A30K (16.3), A30V (39.2), Y93H (37.2)	A30K (99.9), Y93H (99.8)	A30K (99.8), Y93H (19.6)
	8	N	R	None	None	None	None	A30G (32.8), Y93H (99.9)	L31F (33.2), Y93H (99.5)
	9^*c*^	N	R	None	Q80K (99.0)	Q80K (99.4)	V31M (99.8)	V31M (99.8), Y93H (99.8)	V31M (99.9), Y93H (99.9)
TE-PRS									
12 wk	10^*b*^	N	R	None	Y56H (90.8), Q168R (83.6)	None	A30K (99.8)	A30K (99.9), Y93H (99.5)	A30K (99.8)
	11	N	BT	A166S (97.0)	Y56H (95.5), A166S (98.3), Q168L (69.0)	A166S (99.8)	A30K (99.7)	A30K (99.8), Y93H (99.5)	A30K (99.8)
	12	N	R	None	None	NA	Y93H (38.7)	L31F (38.9), Y93H (99.3)	Y93H (99.8)
	13	N	R	None	None	None	A30K (99.6)	A30K (99.8), Y93H (99.6)	A30K (99.8), Y93H (99.3)
	14	N	R	None	None	None	Y93H (99.3)	Y93H (99.6)	Y93H (99.8)
16 wk	15	Y	R	A166S (95.1)	None	NA	None	M28G (97.5)	NA
	16	N	R	None	Y56H (99.4), Q168R (99.8)	None	A30K (99.8)	A30K (99.9), Y93H (99.9)	A30K (99.8), Y93H (81.2)
	17	Y	R	None	None	None	None	L31F (99.7), Y93H (99.8)	L31F (30.7), Y93H (99.6)
	18^*b*^	Y	BT	A166S (99.0)	A156G (99.3), A166S (99.2)	A166S (99.8)	None	A30K (99.8), Y93H (99.7)	A30K (99.9), Y93H (98.9)

a TN, treatment-naive; TE-PRS, treatment-experienced to peg-IFN plus RBV with or without sofosbuvir. Seventeen patients were infected with GT3a, and one was infected with GT3b, as indicated.

b Patient had not been adherent to treatment regimen.

c Patient was infected with GT3b.

d Y, yes; N, no.

e BT, breakthrough; R, relapse.

f VF, virologic failure.

g Variants at amino acid positions 36, 43, 54, 55, 56, 80, 155, 156, 166, and 168 in NS3 or 24, 28, 30, 31, 32, 58, 92, and 93 in NS5A, relative to subtype-specific reference sequences at a 15% detection threshold are listed. None, a polymorphism or substitution at one of at the above-listed amino acid positions was not detected; NA, not available.

Treatment-emergent NS3 substitutions Y56H, Q80R, A156G, and Q168(L/R) were observed in 9 GT3a-infected patients, and A166S or Q168R was present at both baseline and posttreatment in 4 of these patients. Seven of the 17 GT3a-infected patients experiencing virologic failure had multiple substitutions in NS3 at the time of failure. Treatment-emergent substitutions in NS3 remained detectable in 11.1% (1/9) of GT3a-infected patients at posttreatment week 24. Treatment-emergent Y56H, Q80R, and A156G substitutions in NS3 were not detectable at posttreatment week 24, while treatment-emergent Q168(L/R) substitutions were detectable in 16.7% (1/6) of patients. In the GT3a NS3 chimeric replicon, A156G conferred >1,000-fold resistance; Q168L and Q168R substitutions conferred 13- and 54-fold resistance to glecaprevir, respectively (Table 4). The combination of the Y56H substitution plus Q168R in NS3 resulted in greater reductions in glecaprevir susceptibility. NS3-Q80R in GT3a caused a 21-fold reduction in glecaprevir susceptibility (Table 4). The GT3b-infected patient experiencing virologic failure had a treatment-emergent Q80K substitution in NS3, which remained detectable at posttreatment week 24 (Table 7). A GT3b NS3 replicon was not available for phenotypic analysis.

Treatment-emergent NS5A substitutions M28G, A30(G/K), L31F, and Y93H were observed in 15 of 17 GT3a-infected patients, of which 11 patients had A30K (*n* = 8), Y93H (*n* = 2), or A30K as well as Y93H (*n* = 1) at both baseline and posttreatment. Thirteen of the 17 GT3a-infected patients who experienced virologic failure had multiple substitutions in NS5A at the time of failure, of which the most common were the linked substitution of A30K plus Y93H in NS5A detected in 10 patients. Treatment-emergent NS5A substitutions remained detectable at posttreatment week 24 in 73.3% (10/14) of GT3a-infected patients with available data; a treatment-emergent L31F or Y93H substitution was no longer detectable in four of the patients. In the GT3a NS5A chimeric replicon, neither NS5A-A30K nor the Y93H substitution alone conferred resistance to pibrentasvir, while the combination conferred 69-fold resistance. A GT3a replicon containing NS5A-L31F had poor replication capacity and could not be evaluated. The GT3b-infected patient experiencing virologic failure had treatment-emergent Y93H in NS5A, which remained detectable at posttreatment week 24.

Pibrentasvir had an EC_50_ of 15.6 pM against a GT3b NS5A chimeric wild-type replicon with K30 and M31 in NS5A, which was 24-fold lower than the activity against GT3a NS5A chimeric replicon, and presence of Y93H in this GT3b chimeric replicon background reduced susceptibility to pibrentasvir by 6,336-fold (Table 6). In order to determine whether reduced susceptibility of subtype GT3b is unique to pibrentasvir, daclatasvir and velpatasvir, which are each approved for the treatment of GT3 infection in combination with sofosbuvir, were also evaluated against GT3b. Daclatasvir and velpatasvir were each found to have >40,000-fold-reduced activity against GT3b compared with the activity against the GT3a NS5A chimeric replicon. Similar results were recently reported by D. Smith et al. (31). At amino acid positions important for the activity of NS5A inhibitors, the only differences in the GT3a and GT3b sequences are at positions 30 and 31 (Table S2); therefore, substitutions K30A and M31L were introduced sequentially and in combination into the GT3b replicon to investigate the possible impact of these substitutions on the activity of NS5A inhibitors. The substitution K30A alone or M31L alone increased the susceptibility of GT3b replicon to pibrentasvir to levels comparable to the level against the GT3a replicon, suggesting that the presence of either K30 or M31 in patient isolates accounted for the lower activity of pibrentasvir against the wild-type GT3b replicon. Similar improvement in susceptibility of a GT3b replicon to daclatasvir or velpatasvir required a combination of both K30A and M31L substitutions.

### Impact of baseline polymorphisms on SVR12 in GT3-infected patients.

The impact of baseline polymorphisms on treatment outcome in GT3-infected patients was analyzed by treatment duration, prior treatment experience, and cirrhosis status (Table 8). All patients with baseline polymorphisms at amino acid position 36, 43, 55, or 80 in NS3 or 24, 28, 58, or 92 in NS5A achieved SVR12 and are not shown in Table 8. T54S was detected at baseline in one patient, and a Q168R substitution in NS3 was detected at baseline in two patients experiencing virologic failure, one of whom had not adhered to the treatment regimen (Table 7). The low prevalence of T54S and Q168R polymorphisms (<1%) (Table 5) suggested that T54S or Q168R had little impact on the overall treatment outcome. All patients with NS3-A166T or NS5A-A30(L/M/S/T/V) at baseline achieved SVR12. As delineated below, the baseline polymorphism NS3-A166S, NS5A-A30K, or NS5A-Y93H had an impact on treatment outcome in specific GT3 patient populations and treatment durations.

**TABLE 8:** Comparison of SVR12 rates in the presence and absence of baseline polymorphisms in NS3 or NS5A in GT3-infected patients

Patient group and BP^*a*^	SVR12 rate (%) by prior treatment experience, treatment duration, and BP status^*b*^							
	TN (8 wk)		TN (12 wk)		TE-PRS (12 wk)		TE-PRS (16 wk)	
	With BP	Without BP	With BP	Without BP	With BP	Without BP	With BP	Without BP
No cirrhosis								
NS3								
T54(A/S)	50 (1/2)	97 (174/179)	100 (1/1)	99 (250/253)				
A166S	82 (14/17)*	98 (161/164)*	100 (20/20)	99 (231/234)	80.0 (4/5)	90.9 (40/44)	100 (2/2)	95 (18/19)
A166T	100 (8/8)	97 (167/173)	100 (13/13)	99 (238/241)	100 (3/3)	89 (41/46)		
Q168K	100 (1/1)	97 (174/180)	100 (3/3)	99 (248/251)				
Q168R	(0/1)	97 (175/180)	50 (1/2)*	99 (250/252)*				
NS5A								
A30(L/M/S/T/V)	100 (13/13)	96 (163/169)	93 (13/14)^*c*^	99 (240/242)	100 (1/1)	90 (43/48)	100 (1/1)	95 (19/20)
A30K	78 (14/18)***	99 (161/163)***	93 (13/14)^*c*^	99 (240/242)	25 (1/4)**	96 (43/45)**	(0/1)	100 (20/20)
V31M^c^	100 (2/2)	97 (173/179)	(0/1)*	99 (253/255)*	100 (1/1)	90 (43/48)	100 (1/1)	95 (19/20)
Y93H	100 (10/10)	96 (165/171)	91 (10/11)^*c*^	99 (243/245)	50 (2/4)*	93 (42/45)*		
Compensated cirrhosis								
NS3								
A166S			100 (6/6)	100 (53/53)			60 (3/5)*	98 (42/43)*
A166T			100 (3/3)	100 (56/56)			100 (3/3)	93 (42/45)
Q168K			100 (2/2)	100 (57/57)				
Q168R							100 (1/1)	94 (44/47)
NS5A								
A30(M/R/S/T/V)			100 (4/4)	100 (55/55)			100 (4/4)	93 (41/44)
A30K			100 (1/1)	100 (58/58)				
V31M^*d*^							100 (1/1)	94 (44/47)
Y93H			100 (5/5)	100 (54/54)				

a Polymorphisms relative to subtype-specific reference sequences at a 15% detection threshold at amino acid positions 36, 43, 54, 55, 56, 80, 155, 156, 166, and 168 in NS3 or 24, 28, 30, 31, 32, 58, 92, and 93 in NS5A were included in the analysis. Only positions where baseline polymorphisms were detected in patients experiencing virologic failure are listed. All GT3 subtypes are included in the analysis. BP, baseline polymorphism.

b Values in parentheses are the number of patients with the polymorphism/total number of patients in the group with available sequence. TN, treatment-naive; TE-PRS, treatment-experienced to peg-IFN plus RBV with or without sofosbuvir. *, *P* = 0.05; **, *P* = 0.01; ***, *P* = 0.001 (by Fisher's exact test).

c The patient experiencing virologic failure had A30K, A30V, and Y93H at baseline.

d NS5A-V31M was detected only in GT3b-infected patients.

Among TN patients receiving 8 weeks of treatment, the presence of A166S in NS3 was associated with an SVR12 rate of 82% (14/17), and among TE-PRS cirrhotic patients receiving 16 weeks of treatment, the SVR12 rate in the presence of A166S in NS3 was 60% (3/5) as compared to 98% in the absence of A166S in each of these groups (Table 8). Among the five patients experiencing virologic failure with NS3-A166S at baseline, two had not adhered to the treatment regimen, two did not have the A166S substitution detectable at the time of failure or the follow-up time point, and one had no other treatment-emergent substitutions (Table 7). NS3-A166S did not have an impact on treatment outcome in other treatment durations and patient populations (Table 8). These confounding factors (low adherence, lack of treatment-emergent substitutions, and lack of *in vitro* resistance to glecaprevir) attenuate the impact of baseline NS3-A166S on treatment outcome.

Among TN patients receiving 8 weeks of treatment and TE-PRS patients receiving 12 weeks of treatment, numerically lower SVR12 rates were observed in the subset of patients with baseline NS5A-A30K. The overall prevalence of NS5A-A30K in GT3-infected patients in these studies was 6.3% (39/615) (Table 5), similar to the prevalence observed in other studies (32); however, among TN noncirrhotic patients, the prevalence of A30K in the 8-week treatment arm was 2-fold higher than that in the 12-week treatment arm (9.9% versus 5.4%, respectively). Among this subpopulation with baseline NS5A-A30K, there was a numerical difference in the SVR12 rates between those receiving 8 and 12 weeks of treatment (78% [14/18] versus 93% [13/14], respectively) that was driven by four patients with virologic failure in the 8-week arm, one of whom had not been adherent to treatment regimen and one patient in the 12-week arm who had both A30K and Y93H at baseline. Among TE-PRS noncirrhotic patients receiving 12 weeks of treatment, the overall SVR12 rate was 90% (44/49) (Table 1), and the SVR12 rate was 25.0% (1/4) in the presence of NS5A-A30K, 50% (2/4) in the presence of NS5A-Y93H, and 100% (41/41) in the absence of A30K or Y93H (Table 8). A 16-week regimen in this patient population resulted in an SVR12 rate of 96% (Table 1); however, the impact of the A30K or Y93H substitution is unclear due to the low prevalence of the polymorphism in this arm of the study.

It should be noted that in GT3b, K30 in NS5A is the most common amino acid detected at this position and is present in the GT3b reference sequence; K30 in GT3b thus was not considered a polymorphism in this analysis. K30 in NS5A was present in 83.3% (5/6) of the GT3b-infected patients, and 4 of these 5 patients achieved SVR12, as did the single GT3b-infected patient with M30 in NS5A.

Overall, baseline polymorphisms in NS3 and/or NS5A did not have an impact on treatment outcome, with the exception of GT3-infected TE-PRS patients treated for 12 weeks.

## DISCUSSION

The efficacy and safety of the once-daily, RBV-free, glecaprevir/pibrentasvir regimen was evaluated in HCV-infected patients in eight global registrational phase 2 and 3 studies: SURVEYOR-1 and -2, ENDURANCE-1, -2, -3, and -4, and EXPEDITION-1 and -4 (20–28). Phylogenetic analysis of HCV sequences obtained from 2,173 patients in the ITT population with available baseline NS3/4A and/or NS5A sequences identified 37 subtypes. The pooled overall SVR12 rate was 98% in 2,256 patients in the ITT population, with a <1% virologic failure rate (22 of 2,256 patients).

Baseline polymorphisms in NS3 (Q80K) or NS5A (especially Y93H) are associated with reduced treatment efficacy for multiple DAA regimens, including ombitasvir/paritaprevir/ritonavir with or without dasabuvir, grazoprevir/elbasvir, and ledipasvir/sofosbuvir, requiring longer treatment durations or the addition of RBV (14). The prevalence of baseline polymorphisms in NS3 or NS5A that confer resistance to glecaprevir or pibrentasvir was assessed in the pooled analysis in patients infected with GT1 to GT6. Baseline polymorphisms at NS3 amino acid position A156, which caused the greatest reductions (>100-fold) in susceptibility to glecaprevir in most genotypes, or those at position D168 that caused >30-fold resistance to glecaprevir, such as D168(F/Y) in GT1a or D168(A/G/H/V/Y) in GT6a, were not detected in any patient (19). Q168R in NS3 in GT3a, which confers 54-fold resistance to glecaprevir, was rarely detected at baseline (0.7%) (19). Q80R in NS3 in GT3a, which causes a 21-fold reduction in glecaprevir susceptibility *in vitro*, was not detected at baseline in any of the GT3-infected patients. Other polymorphisms detected at baseline at amino acid position 36, 43, 54, 55, 56, 80, 155, 166, or 170 in NS3 generally did not confer resistance to glecaprevir (19).

The majority of individual amino acid substitutions associated with resistance to other HCV NS5A inhibitors at amino acid position 24, 28, 30, 31, 58, 92, or 93 in NS5A did not confer resistance to pibrentasvir (18). Substitutions at NS5A amino acid position 93 are known to confer high levels of resistance to NS5A inhibitors daclatasvir, elbasvir, ledipasvir, ombitasvir, and velpatasvir and have influenced treatment outcome in regimens containing these NS5A inhibitors in specific genotypes and patient populations (14). NS5A-Y93 substitutions remained susceptible to pibrentasvir *in vitro* and were detected at a prevalence of around 5% in GT1-, GT3-, or GT6-infected patients.

Among HCV-infected patients treated with the pan-genotypic sofosbuvir/velpatasvir regimen, baseline NS5A polymorphisms had no effect on treatment outcome in non-GT3-infected patients (14). The SVR12 rates of sofosbuvir/velpatasvir among cirrhotic GT3-infected patients were lower in the presence of NS5A baseline polymorphisms, especially Y93H. Overall efficacy of sofosbuvir/velpatasvir for GT3-infected patients was lower for peg-IFN treatment-experienced patients (90%, 64/71) than for treatment-naive patients (98%, 200/204); however, the data set for the treatment-experienced subgroup was too limited for a robust analysis of impact of baseline polymorphisms (SVR12 rates of 83% [5/6] and 90% [59/65] for those with and without baseline NS5A polymorphisms, respectively) (14). Sofosbuvir/velpatasvir/voxilaprevir is approved in Europe for the treatment of DAA treatment-naive patients infected with GT1 to GT6 with or without cirrhosis for treatment duration of 8 or 12 weeks (33). This regimen had an overall SVR12 rate of 95% largely due to the lower SVR12 rate of 92% among GT1a-infected patients, specifically those with baseline Q80K in NS3, but other NS3 or NS5A baseline polymorphisms did not seem to impact treatment outcome (14).

Among patients treated with the glecaprevir/pibrentasvir regimen, baseline polymorphisms in NS3 or NS5A, including the prevalent NS3-Q80K polymorphism in GT1a, had no impact on treatment outcome, regardless of treatment duration, prior treatment history, or cirrhosis status for patients infected with GT1, -2, -4, -5, or -6. The SVR12 rate in the modified-ITT population of TN and TE-PRS GT1-, GT2-, GT3-, GT4-, GT5-, or GT6-infected patients without cirrhosis receiving 8 weeks or 12 weeks of treatment was >99% (632/635) and 100% (802/802), respectively, and >99% (180/181) in patients with cirrhosis receiving 12 weeks of treatment. The glecaprevir/pibrentasvir regimen has received marketing approvals in many countries for the treatment of TN and TE-PRS GT1-, GT2-, GT3-, GT4-, GT5-, or GT6-infected patients for durations of 8 weeks or 12 weeks in patients without cirrhosis or with compensated cirrhosis, respectively. Though the number of GT5- and GT6-infected patients receiving the recommended regimen was low in the phase 2 and 3 studies; high SVR12 rates were demonstrated in the phase 3b study ENDURANCE-56 that enrolled 84 GT5- and GT6-infected patients (34).

Baseline polymorphisms in NS3 or NS5A did not have an impact on SVR12 in GT3-infected patients treated with glecaprevir/pibrentasvir, except in TE-PRS patients receiving 12 weeks of treatment. Among TE-PRS noncirrhotic patients receiving 12 weeks of treatment, the SVR12 rates in patients with baseline NS5A-A30K or NS5A-Y93H were lower than the rates in patients without either polymorphism. The approved duration of glecaprevir/pibrentasvir treatment for GT3-infected TE-PRS patients is 16 weeks, and due to the low prevalence of NS5A-A30K or NS5A-Y93H, the impact of either of these substitutions in the population receiving 16 weeks of treatment remains to be confirmed with additional data collected from real-world treatment. The presence of NS5A-A30K was associated with a numerically lower SVR12 rate in TN noncirrhotic patients receiving 8 weeks of treatment than in those receiving 12 weeks of treatment (78% [14/18] versus 93% [13/14]) although the overall SVR12 rates in TN noncirrhotic patients receiving 8 weeks and 12 weeks of treatment were comparable (95% [177/183] versus 96% [258/261], respectively). Additionally, the prevalence of A30K in GT3-infected patients in the 8-week treatment arm was 2-fold higher than that in the 12-week treatment arm (9.9% versus 5.4%, respectively). Assuming a linear relationship between A30K prevalence and virologic failure rate and equivalent prevalences of A30K in the 12-week and 8-week arms (9.9%), there was a <1% difference in SVR12 rates between the 8- and 12-week durations. Consistent with these observations, the glecaprevir/pibrentasvir regimen has received marketing approval in many countries for treatment durations of 8 weeks in noncirrhotic TN GT3-infected patients, of 12 weeks in TN GT3-infected patients with compensated cirrhosis, and of 16 weeks in TE-PRS GT3-infected patients irrespective of cirrhosis status.

Ten of 18 GT3-infected virologic failures, 9 of whom had a preexisting A30K substitution at baseline, had A30K in combination with Y93H in NS5A at the time of failure. Although neither A30K nor Y93H in NS5A alone conferred resistance to pibrentasvir in GT3a, the combination conferred 69-fold resistance in the GT3a replicon. In patients with a preexisting A30K substitution, acquiring Y93H requires a single nucleotide change, whereas in patients with preexisting Y93H, the acquisition of an A30K amino acid substitution requires a 2-nucleotide change. This may explain the detection of A30K in combination with Y93H at the time of failure in patients with preexisting A30K experiencing virologic failure and the relatively higher impact of the A30K polymorphism than of Y93H on treatment outcome. NS5A-Y93H in GT3a confers 3,733- and 724-fold resistances to daclatasvir and velpatasvir, respectively, while NS5A-A30K confers 117-fold and 50-fold resistances to daclatasvir and velpatasvir, respectively (14). The majority of patients experiencing virologic failure with regimens containing daclatasvir or velpatasvir had only a Y93H substitution at the time of failure, and baseline Y93H was associated with lower SVR12 rates in some patient populations (35, 36).

Consistent with the low prevalence of GT3b in non-Asian countries, only 6 GT3b-infected patients were enrolled in the studies included in this pooled analysis. All 6 GT3b-infected patients had M31 in NS5A, and K30 in NS5A was present in samples obtained from 83% (5/6) of the GT3b-infected patients; 4 of the 5 patients with K30 achieved SVR12. Pibrentasvir had 24-fold lower activity against GT3b wild-type chimeric replicon containing K30 and M31 in NS5A. Of note, the activities of daclatasvir and velpatasvir were >40,000-fold lower in GT3b than in GT3a replicon. These differences in activities against GT3a and GT3b chimeric replicons were attributed to the presence of NS5A-K30 and/or NS5A-M31 in GT3b compared to NS5A-A30 and NS5A-L31 in GT3a.

Seventeen of the 22 patients receiving glecaprevir/pibrentasvir who experienced virologic failure in the registrational phase 2 and 3 studies (1 GT1a, 2 GT2a, 13 GT3a, and 1 GT3b infections) were enrolled in the MAGELLAN-3 retreatment study, where the patients received glecaprevir/pibrentasvir plus sofosbuvir plus RBV for 12 or 16 weeks; all achieved SVR12 (37). The glecaprevir/pibrentasvir regimen has been approved in some countries for the treatment of protease inhibitor-experienced patients without prior experience with an NS5A inhibitor (12-week treatment duration) or of NS5A inhibitor-experienced patients without prior experience with a protease inhibitor (16-week treatment duration) (38). The combination regimen of sofosbuvir/velpatasvir/voxilaprevir administered for 12 weeks has been approved for treatment of DAA-experienced patients, including those who previously failed an NS5A inhibitor-containing regimen (39).

In summary, eight global phase 2 and 3 registrational studies evaluated the glecaprevir/pibrentasvir regimen in 2,256 TN and TE-PRS patients without cirrhosis or with compensated cirrhosis and with or without severe renal impairment. The overall virologic failure rate was low (<1%). With the label-recommended treatment durations, high SVR12 rates (98.7%, 1,104/1,118) were achieved in all HCV genotypes/subtypes irrespective of the presence of baseline polymorphisms in NS3 and/or NS5A, and baseline resistance testing is not recommended in the current American Association for the Study of Liver Diseases (AASLD) and European Association for the Study of the Liver (EASL) guidelines (40, 41).

## MATERIALS AND METHODS

### Clinical studies.

The pooled resistance analyses included available HCV sequence data from TN and TE-PRS patients in arms that were administered glecaprevir at 300 mg once a day (QD) and pibrentasvir at 120 mg QD (without RBV) in phase 2 or 3 studies. Description of each study design, randomization procedures, and efficacy and safety analyses were previously described. They are outlined briefly below.

- ENDURANCE-1 (ClinicalTrials.gov identifier NCT02604017) was a phase 3, open-label, multicenter study where HCV GT1-monoinfected or HIV-1/HCV GT1-coinfected TN or TE-PRS patients without cirrhosis were randomly assigned in a 1:1 ratio to receive glecaprevir/pibrentasvir for 8 or 12 weeks (27).
- ENDURANCE-2 (ClinicalTrials.gov identifier NCT02640482) was a phase 3, randomized, double-blinded, placebo-controlled, multicenter study. HCV GT2-infected, noncirrhotic TN or TE-PRS patients were randomized in a 2:1 ratio to receive either glecaprevir/pibrentasvir or placebo for 12 weeks during the double-blind treatment period. Patients randomized to the placebo arm received open-label glecaprevir/pibrentasvir for 12 weeks after completion of placebo (25).
- ENDURANCE-3 (ClinicalTrials.gov identifier NCT02640157) had an active-controlled, partially randomized design according to which TN patients without cirrhosis were randomly assigned in a 2:1 ratio to receive either glecaprevir/pibrentasvir or 400 mg of sofosbuvir plus 60 mg of daclatasvir for 12 weeks. After additional phase 2 data that supported the efficacy of an 8-week treatment duration became available, a subsequent protocol amendment enabled the nonrandom assignment of TN noncirrhotic patients into a third group for 8 weeks of treatment with glecaprevir/pibrentasvir (27).
- ENDURANCE-4 (ClinicalTrials.gov identifier NCT02636595) was an open-label, multicenter, single-arm phase 3 study that evaluated 12 weeks of glecaprevir/pibrentasvir in noncirrhotic TN or TE-PRS patients with HCV GT4, GT5, or GT6 infection (25).
- EXPEDITION-1 (ClinicalTrials.gov identifier NCT02642432) was an open-label, multicenter, single-arm phase 3 study that assessed 12 weeks of glecaprevir/pibrentasvir in TN or TE-PRS patients with GT1, -2, -4, -5, or -6 infection and compensated cirrhosis (21, 23).
- EXPEDITION-4 (ClinicalTrials.gov identifier NCT02651194) was on open-label, single-arm, multicenter phase 3 study that assessed 12 weeks of glecaprevir/pibrentasvir in GT1-, GT2-, GT3-, GT4-, GT5-, or GT6-infected TN or TE-PRS patients with or without cirrhosis and severe renal impairment (chronic kidney disease [CKD] stages 4 and 5) (28).
- SURVEYOR-2 (ClinicalTrials.gov identifier NCT02243293) part 2 was a phase 2 partially randomized, open-label, multicenter study that assessed glecaprevir plus pibrentasvir in noncirrhotic TN and peg-IFN/RBV-experienced GT2-infected patients for 8 weeks, noncirrhotic TN GT3-infected patients for 8 weeks, and noncirrhotic GT3-infected peg-IFN/RBV-experienced patients for 12 weeks. TN GT3-infected patients with cirrhosis were randomized in a 1:1 ratio to receive glecaprevir plus pibrentasvir with or without RBV for 12 weeks (patients receiving RBV were excluded from the pooled resistance analysis). Part 3 of SURVEYOR-2 was a phase 3, partially randomized, open-label, multicenter study that assessed glecaprevir/pibrentasvir in GT3-infected patients with or without cirrhosis. TN patients with cirrhosis received 12 weeks of treatment, TE-PRS patients without cirrhosis were randomized in a 1:1 ratio to receive 12 or 16 weeks of treatment, and TE-PRS patients with cirrhosis received 16 weeks of treatment. SURVEYOR-2 part 4 was an open-label, multicenter, single-arm, phase 3 study that evaluated 8 weeks of glecaprevir/pibrentasvir in noncirrhotic patients with HCV GT2, -4, -5, or -6 infection (20, 23–25).
- SURVEYOR-1 (ClinicalTrials.gov identifier NCT02243280) part 2 was a phase 2, open-label, multicenter study that assessed glecaprevir plus pibrentasvir in TN or peg-IFN/RBV-experienced patients without cirrhosis. GT1-infected patients received 8 weeks of treatment, and patients infected with GT4 to GT6 received 12 weeks of treatment (23).

All patients provided written, informed consent to participate, and the studies were conducted in accordance with the ethical guidelines of the Declaration of Helsinki and the International Conference on Harmonization Good Clinical Practice Guidelines. The study was approved by an institutional review board of each study site prior to the initiation of any screening or study-specific procedures.

### Sample processing.

Methods for extraction of HCV RNA and amplification of NS3/4A and NS5A regions were described previously (42, 43). Only samples with ≥1,000 IU/ml of HCV RNA were amplified in order to reduce the chances of oversampling bias. For samples with ≤50,000 IU/ml of HCV RNA, reverse transcription-PCRs (RT-PCRs) were done in triplicate, and the products were pooled prior to their use as a template for nested PCR. Nested PCR products encompassing the genes encoding full-length NS3/4A or NS5A were analyzed by next-generation sequencing (NGS), analysis was performed by DDL Diagnostic Laboratory (Rijswijk, Netherlands) (42) or by Monogram Biosciences (San Francisco, CA) using proprietary methods. PCR primer information and cycling conditions are provided in Tables S4 and S5 in the supplemental material.

### HCV genotype and subtype classification.

For each sample analyzed by NGS, a consensus sequence was generated for each target gene from the NGS nucleotide sequences, with an ambiguity setting of 0.25. Nucleotide sequences for NS3/4A and NS5A were aligned using the MAFFT sequence alignment method (44). Phylogenetic trees were constructed using the neighbor-joining tree-building method with the HKY85 nucleotide substitution model (45, 46). Reliability of the tree topology was examined using bootstrap analysis, and 1,000 bootstrapping replicates were utilized to generate a consensus tree with a 50% threshold cutoff. Nucleotide alignments and phylogenetic trees were generated using Geneious software (Biomatters, Ltd., Auckland, New Zealand). The final HCV subtype assignment was determined by consensus between NS3/4A and NS5A phylogenetic analysis results. If sequences were not available for phylogenetic analyses, subtype assignment by LiPA, version 2.0, or Sanger sequencing was utilized.

### Resistance analyses.

Analysis was conducted on a modified-ITT population, in which patients who did not achieve SVR12 due to reasons unrelated to efficacy, such as premature discontinuation, missing HCV RNA results in SVR12 window, or reinfection, were excluded from the analysis. Treatment adherence was calculated as the percentage of tablets taken (determined by pill counts at study visits from weeks 4, 8, 12 [where applicable], and 16 [where applicable]) relative to the total expected number of tablets, where adherence needed to be between 80% and 120% at each 4-week dispensation interval (thus, values below 80% and above 120% were considered nonadherent).

Analyses were grouped by HCV subtype, treatment duration, prior HCV treatment experience, or cirrhosis status and broadly included the following analyses: (i) prevalence of polymorphisms at baseline at amino acid positions important for the NS3/4A protease and NS5A inhibitor class at a 15% NGS detection threshold relative to the appropriate subtype-specific reference sequence, as described in Tables S1 and S2 in the supplemental material; (ii) impact of baseline polymorphisms on treatment response, with SVR12 rates compared in patients with or without baseline polymorphisms by Fisher's exact test; and (iii) analysis of baseline polymorphisms and treatment-emergent substitutions relative to baseline sequence in patients experiencing virologic failure. A polymorphism was defined as an amino acid at a position in the HCV sequence from a baseline sample that differs from the amino acid at that position in the appropriate subtype-specific reference sequence. A treatment-emergent substitution is defined as an amino acid at a position in the HCV sequence that was not present at baseline and was observed at a postbaseline time point.

### Antiviral activity against a panel of NS3 or NS5A amino acid substitutions.

The methods for assessing the measurement of the effects of individual amino acid substitutions on the activity of an inhibitor in HCV replicon cell culture assays were described previously (18, 19, 47). For HCV GT3b, a consensus sequence for NS5A was derived from an alignment of 10 GT3b-infected patient sequences and the GT3b HCV-Tr sequence (GenBank accession number D49374). The NS5A GT3b consensus sequence encompassing amino acids 1 to 187 was generated as a synthetic gene (Integrated DNA Technologies [IDT], Coralville, IA) and ligated into an HCV GT2a JFH1 strain subgenomic transient replicon vector containing a luciferase reporter gene (48) in place of the corresponding region from GT2a JFH1 (G. Schnell, P. Krishnan, R. Tripathi, J. Beyer, T. Reisch, M. Irvin, T. Dekhtyar, L. Lu, T. Ng, W. Xie, T. Pilot-Matias, and C. Collins, submitted for publication). NS3 and NS5A substitutions were each introduced into the subtype-specific subgenomic replicon plasmid using a Change-IT Multiple Mutation site-directed mutagenesis kit (Affymetrix, Santa Clara, CA), or synthetic DNA constructs encoding NS3 and NS5A substitutions (Integrated DNA Technologies, Coralville, IA) were inserted into subtype-specific subgenomic replicon plasmid. In a transient assay, the replicon RNA containing the substitutions was transfected via electroporation into a Huh7 cell line. Glecaprevir, pibrentasvir, and daclatasvir were synthesized at AbbVie. Velpatasvir was purchased from eNovation Chemicals (Bridgewater, NJ). The luciferase activity in the cells was measured using an EnVision Multilabel Plate Reader (Perkin-Elmer, Waltham, MA). The EC_50_s were calculated using nonlinear regression curve fitting to the four-parameter logistic equation in Prism, version 5, software (GraphPad Software, Inc., La Jolla, CA). Mean EC_50_s and standard deviations were calculated from at least three independent experiments.

## Supplementary Material

Supplemental file 1
