# Supplementary material for: Pooled Resistance Analysis in Patients with Hepatitis C Virus Genotype 1 to 6 Infection Treated with Glecaprevir-Pibrentasvir in Phase 2 and 3 Clinical Trials
Source: Antimicrob Agents Chemother. 2018 Sep 24;62(10):e01249-18. doi: 10.1128/AAC.01249-18 (PMC6153825; doi:10.1128/AAC.01249-18)
Supplement: Supplemental file 1 [file zac010187522s1.pdf]

Pooled Resistance Analysis in HCV Genotype 1-6 Infected Patients Treated With Glecaprevir/Pibrentasvir in Phase 2 and 3 Clinical Trials

Preethi Krishnan[#], Tami Pilot-Matias, Gretja Schnell, Rakesh Tripathi, Teresa I. Ng, Thomas Reisch, Jill Beyer, Tatyana Dekhtyar, Michelle Irvin, Wangang Xie, Lois Larsen, Federico J. Mensa, and Christine Collins

Research & Development, AbbVie Inc., North Chicago, IL, 60064

[#] Address correspondence to preethi.krishnan@abbvie.com

SUPPLEMENTAL TABLES

Supplemental Table S1. Most prevalent amino acids at each key position in NS3 in subtype-specific reference sequences

			Amino Acid Positions in NS3									
GT	GenBank Accession	Strain	36	43	54	55	56	80	155	156	166	168
1a	NC_004102	H77	V	F	T	V	Y	Q	R	A	A	D
1b	AJ238799	Con1	V	F	T	V	Y	Q	R	A	A	D
1e	KJ439769	QC172	L	F	S	V	Y	Q	R	A	A	D
1g	AM910652	1804	V	F	S	V	Y	Q	R	A	A	D
2a	AB047639	JFH-1	L	F	T	V	Y	G	R	A	S	D
2b	D10988	HC-J8	L	F	T	V	Y	G	R	A	S	D
2c	D50409	BEBE1	L	F	T	V	F	G	R	A	S	D
2i	DQ155561	D54	L	F	T	V	F	G	R	A	S	D
2k	AB031663	VAT96	L	F	T	V	F	G	R	A	S	D
2l	KC197235	MRS89	L	F	T	V	Y	G	R	A	A	D
2q	FN666429	852	L	F	T	V	F	G	R	A	S	D
2t	KC197238	MRS40	L	F	T	V	F	G	R	A	A	D
3a	GU814263	S52	L	F	T	V	Y	Q	R	A	A	Q
3b	D49374	HCV-Tr	L	F	T	V	Y	Q	R	A	A	Q
3i	FJ407092	IND-HCV-3i	L	F	T	V	Y	Q	R	A	A	Q
4a	GU814265	ED43	L	F	T	V	Y	Q	R	A	A	D
4c	FJ462436	QC381	L	F	T	V	Y	Q	R	A	A	D
4d	FJ462437	QC382	L	F	T	V	Y	Q	R	A	A	D
4f	EF589161	IFBT88	L	F	T	V	Y	Q	R	A	A	D
4g	FJ462432	QC193	L	F	T	V	Y	Q	R	A	A	D
4k	FJ462438	QC383	L	F	T	V	Y	Q	R	A	A	D
4m	FJ462433	QC249	L	F	T	V	Y	Q	R	A	A	D
4n	FJ462441	QC97	L	F	T	V	Y	Q	R	A	A	D
4o	FJ462440	QC93	L	F	T	V	Y	Q	R	A	A	D
4q	FJ462434	QC262	L	F	T	V	Y	Q	R	A	A	D
4r	FJ462439	QC384	L	F	T	V	Y	Q	R	A	A	D
4t	FJ839869	QC155	L	F	T	V	Y	Q	R	A	A	D
4v	HQ537008	CYHCV048	L	F	T	V	Y	Q	R	A	A	D
5a	AF064490	SA13	L	F	T	V	F	K	R	A	A	D
6a	Y12083	EUHK2	V	F	T	V	Y	L	R	A	S	D
6e	DQ314805	GX004	V	F	T	V	Y	Q	R	A	A	D
6h	D84265	VN004	V	F	T	V	Y	Q	R	A	S	D
6l	EF424628	537796	V	F	T	V	Y	Q	R	A	S	D

			Amino Acid Positions in NS3									
**GT**	**GenBank Accession**	**Strain**	**36**	**43**	**54**	**55**	**56**	**80**	**155**	**156**	**166**	**168**
6p	EF424626	QC216	V	F	T	V	Y	Q	R	A	A	D
6q	EF424625	QC99	I	F	T	V	Y	Q	R	A	A	D
6r	EU408328	QC245	V	F	T	V	F	Q	R	A	A	D
6t	EU246939	D49	L	F	T	V	F	Q	R	A	A	D

Supplemental Table S2. Most prevalent amino acids at each key position in NS5A in subtype-specific reference sequences

			Amino Acid Position in NS5A							
**GT**	**GenBank Accession**	**Strain**	**24**	**28**	**30**	**31**	**32**	**58**	**92**	**93**
1a	NC_004102	H77	K	M	Q	L	P	H	A	Y
1b	AJ238799	Con1	Q	L	R	L	P	P	A	Y
1e	KJ439769	QC172	R	M	Q	L	P	P	T	Y
1g	AM910652	1804	S	L	R	L	P	P	A	F
2a	AB047639	JFH-1	T	F	K	L	P	P	C	Y
2b	D10988	HC-J8	S	L	K	M	P	P	C	Y
2c	D50409	BEBE1	S	F	R	L	P	P	C	Y
2i	DQ155561	D54	S	F	K	M	P	P	C	Y
2k	AB031663	VAT96	S	L	K	M	P	P	C	Y
2l	KC197235	MRS89	S	L	K	L	P	P	S	Y
2q	FN666429	852	S	L	K	M	P	P	C	Y
2t	KC197238	MRS40	S	L	K	L	P	P	C	Y
3a	GU814263	S52	S	M	A	L	P	P	E	Y
3b	D49374	HCV-Tr	S	M	K	V	P	P	E	Y
3i	FJ407092	IND-HCV-3i	S	M	K	L	P	P	E	Y
4a	GU814265	ED43	K	L	L	M	P	P	A	Y
4c	FJ462436	QC381	K	L	R	M	P	P	A	Y
4d	FJ462437	QC382	K	L	R	M	P	T	A	Y
4f	EF589161	IFBT88	K	L	Q	M	P	P	A	Y
4g	FJ462432	QC193	K	L	L	M	P	P	A	H
4k	FJ462438	QC383	K	L	R	M	P	P	A	Y
4m	FJ462433	QC249	K	L	S	M	P	P	A	Y
4n	FJ462441	QC97	K	L	R	M	P	T	A	Y
4o	FJ462440	QC93	K	M	T	M	P	P	A	Y
4q	FJ462434	QC262	K	L	R	M	P	P	A	Y
4r	FJ462439	QC384	K	I	R	L	P	P	A	Y
4t	FJ839869	QC155	K	L	R	M	P	P	A	Y
4v	HQ537008	CYHCV048	K	L	R	M	P	P	A	Y
5a	AF064490	SA13	Q	L	Q	L	P	P	A	T
6a	Y12083	EUHK2	Q	F	R	L	P	T	A	T
6e	DQ314805	GX004	K	V	S	L	P	P	A	T
6h	D84265	VN004	K	V	A	L	P	P	A	T
6l	EF424628	537796	K	V	A	L	P	P	A	T
6p	EF424626	QC216	K	V	S	L	P	P	A	T
6q	EF424625	QC99	K	V	S	L	P	P	A	T

			Amino Acid Position in NS5A							
**GT**	**GenBank Accession**	**Strain**	**24**	**28**	**30**	**31**	**32**	**58**	**92**	**93**
6r	EU408328	QC245	K	G	A	L	P	P	A	T
6t	EU246939	D49	K	V	S	L	P	G	A	T

Supplemental Table S3. Prevalence of baseline polymorphisms in NS3 and/or NS5A in patients infected with HCV GT 1, 2, 4, 5, or 6 in phase 2 and 3 registrational studies

HCV Subtype, Reference Genbank Accession number, Strain	NS3		NS5A	
	Baseline Polymorphisms[a]	% (n/N)	Baseline Polymorphisms[a]	% (n/N)
Total GT1	Any	38.9 (329/846)	Any	26.8 (226/842)
GT1a, NC_004102, H77	Any	48.7 (187/384)	Any	20.5 (78/380)
	V36A/L/M	3.4 (13/384)	K24Q/R	1.6 (6/380)
	T54S/Y	2.1 (8/384)	M28I/L/T/V	8.7 (33/380)
	V55A/I/P	4.9 (19/384)	Q30H/L/R	1.8 (7/380)
	Y56F	0.3 (1/384)	L31M	2.1 (8/380)
	Q80K/L/M/R	37.5 (144/384)	H58D/L/N/P/Q/R/Y	6.6 (25/380)
	R155K	1.0 (4/384)	A92P	0.3 (1/380)
	D168E	1.0 (4/384)	Y93C/F/H/N/S	2.1 (8/380)
GT1b, AJ238799, Con1	Any	30.8 (142/461)	Any	31.9 (147/461)
	V36I/L	1.5 (7/461)	Q24K/R	1.1 (5/461)
	T54S/Y	1.7 (8/461)	L28M	2.4 (11/461)
	V55A/P	0.7 (3/461)	R30K/L/M/Q	4.8 (22/461)
	Y56F/L	31.9 (147/461)	L31I/M	4.6 (21/461)
	Q80H/K/L/R	5.0 (23/461)	P58A/H/L/Q/R/S/T	9.5 (44/461)
	D168E	0.2 (1/461)	A92E/I/T/V	7.6 (35/461)
			Y93F/H/S	8.2 (38/461)
GT1g, AM910652, 1804	Any	(0/1)	Any	100 (1/1)
			S24R	100 (1/1)
			R30Q	100 (1/1)
Total GT2	Any	13.1 (52/398)	Any	79.8 (331/415)

HCV Subtype, Reference Genbank Accession number, Strain	NS3		NS5A	
	Baseline Polymorphisms[a]	% (n/N)	Baseline Polymorphisms[a]	% (n/N)
GT2a, AB047639, JFH-1	Any	10.6 (11/104)	Any	94.5 (103/109)
	L36M	1.9 (2/104)	T24A/S	9.2 (10/109)
	Y56F	6.7 (7/104)	F28C/L/V	3.7 (4/109)
	D168E	1.9 (2/104)	K30R	1.8 (2/109)
			L31M	93.6 (102/109)
			L31V	0.9 (1/109)
			P58S	2.8 (3/109)
			C92S	2.8 (3/109)
GT2b, D10988, HC-J8	Any	17.4 (41/236)	Any	71.8 (168/234)
	V55I	0.4 (1/236)	L28F	3.4 (8/234)
	Y56F/H/L	16.9 (40/236)	K30R	0.4 (1/234)
	D168V	0.4 (1/236)	M31I/V	1.3 (3/234)
			M31L	67.1 (157/234)
			P58A/S	4.7 (11/234)
			C92S	1.3 (3/234)
GT2c, D50409, BEBE1	Any	(0/48)	Any	90.9 (50/55)
			S24A	1.8 (1/55)
			F28C	38.2 (21/55)
			R30K	89.1 (49/55)
			L31F/M	20.0 (11/55)
			P58A	1.8 (1/55)
			C92W	1.8 (1/55)
GT2i, DQ155561, D54	Any	(0/6)	Any	42.9 (3/7)
			F28L	14.3 (1/7)

HCV Subtype, Reference Genbank Accession number, Strain	NS3		NS5A	
	Baseline Polymorphisms[a]	% (n/N)	Baseline Polymorphisms[a]	% (n/N)
			K30R	28.6 (2/7)
GT2k, AB031663, VAT96	Any	-	Any	(0/1)
GT2l, KC197235, MRS89	Any	(0/2)	Any	33.3 (1/3)
			S92C	33.3 (1/3)
GT2q, FN666429, 852	Any	(0/1)	Any	100 (1/1)
			S24T	100 (1/1)
			L28F	100 (1/1)
GT2t, KC197238, MRS40	Any	-	Any	100 (1/1)
			L28F	100 (1/1)
			P58T	100 (1/1)
Total GT4	Any	4.9 (8/164)	Any	49.7 (80/161)
GT4a, GU814265, ED43	Any	5.1 (4/79)	Any	26.6 (21/79)
	T54S	5.1 (4/79)	L28M/V	12.7 (10/79)
			L30R	10.1 (8/79)
			P58L/S/T	6.3 (5/79)
GT4c, FJ462436, QC381	Any	(0/1)	Any	(0/1)
GT4d, FJ462437, QC382	Any	3.9 (2/51)	Any	82.4 (42/51)
	Q80K	2.0 (1/51)	M31L/V	3.9 (2/51)
	D168E	2.0 (1/51)	T58A/L/P	82.4 (42/51)
GT4f, EF589161, IFBT88	Any	50.0 (1/2)	Any	100 (2/2)
	Y56F	50.0 (1/2)	Q30R	100 (2/2)
			M31L	50.0 (1/2)
			P58T	50.0 (1/2)
GT4g, FJ462432, QC193	Any	(0/2)	Any	100 (1/1)

HCV Subtype, Reference Genbank Accession number, Strain	NS3		NS5A	
	Baseline Polymorphisms[a]	% (n/N)	Baseline Polymorphisms[a]	% (n/N)
			L30C	100 (1/1)
			M31L	100 (1/1)
			H93Y	100 (1/1)
GT4g/4k, FJ462438, QC383	Any	(0/1)	Any	100 (1/1)
			R30C	100 (1/1)
			M31L	100 (1/1)
GT4k, FJ462438, QC383	Any	11.1 (1/9)	Any	87.5 (7/8)
	D168E	11.1 (1/9)	M31L	87.5 (7/8)
GT4m, FJ462433, QC249	Any	(0/1)	Any	(0/1)
GT4n, FJ462441, QC97	Any	(0/1)	Any	(0/1)
GT4o, FJ462440, QC93	Any	(0/4)	Any	33.3 (1/3)
			T30A	33.3 (1/3)
			M31L	33.3 (1/3)
GT4q, FJ462434, QC262	Any	(0/2)	Any	(0/2)
GT4r, FJ462439, QC384	Any	(0/7)	Any	71.4 (5/7)
			I28M/V	71.4 (5/7)
			R30H	14.3 (1/7)
			Y93H	14.3 (1/7)
GT4t, FJ839869, QC155	Any	(0/1)	Any	(0/1)
GT4v, HQ537008, CYHCV048	Any	(0/3)	Any	(0/3)
GT5a, AF064490, SA13	Any	45.2 (14/31)	Any	12.9 (4/31)
	V55I	3.2 (1/31)	Q30R/L	6.5 (2/31)
	F56Y	3.2 (1/31)	L31F	3.2 (1/31)
	D168E	41.9 (13/31)	A92S	3.2 (1/31)

HCV Subtype, Reference Genbank Accession number, Strain	NS3		NS5A	
	**Baseline Polymorphisms[a]**	**% (n/N)**	**Baseline Polymorphisms[a]**	**% (n/N)**
Total GT6	Any	41.2 (14/34)	Any	54.1 (20/37)
GT6a, Y12083, EUHK2	Any	100 (11/11)	Any	63.6 (7/11)
	L80K	100 (11/11)	Q24K/R	18.2 (2/11)
	D168E	9.1 (1/11)	F28L	54.5 (6/11)
GT6e, DQ314805, GX004	Any	13.3 (2/15)	Any	56.3 (9/16)
	V36L	13.3 (2/15)	K24R	12.5 (2/16)
			V28M	37.5 (6/16)
			L31I	6.3 (1/16)
			P58S	18.8 (3/16)
			T93S	12.5 (2/16)
GT6h, D84265, VN004	Any	(0/1)	Any	(0/1)
GT6l, EF424628, 537796	Any	-	Any	(0/1)
GT6p, EF424626, QC216	Any	50.0 (1/2)	Any	50.0 (1/2)
	V36I	50.0 (1/2)	V28M	50.0 (1/2)
GT6q, EF424625, QC99	Any	(0/2)	Any	(0/2)
GT6r, EU408328, QC245	Any	(0/3)	Any	100 (3/3)
			K24R	33.3 (1/3)
			G28A/T	100 (3/3)
GT6t, EU246939, D49	Any	-	Any	(0/1)

a. Polymorphisms relative to subtype-specific reference sequences at amino acid positions 36, 43, 54, 55, 56, 155, 156, 168 in NS3, and 24, 28, 30, 31, 58, 92, 93 in NS5A at 15% detection threshold are listed. 'Any' indicates total number of patients with any polymorphism at signature amino acid positions within each target.

Supplemental Table S4. Amplification of NS3/4A from HCV-infected clinical samples

RT-PCR reaction was conducted using SuperScript III One-Step RT-PCR System with Platinum Taq High Fidelity (Invitrogen) using cycling conditions: cDNA synthesis at 55°C for 30 minutes; denaturation at 94°C for 2 minutes; PCR amplification with 40 cycles of denaturation at 94°C for 2 minutes, annealing at 58°C for 30 seconds, extension at 68°C for 3 minutes; final extension at 68°C for 5 minutes.

Nested PCR reaction was conducted using Platinum Pfx DNA Polymerase (Invitrogen) using cycling conditions: denaturation at 94°C for 5 minutes; PCR amplification with 35 cycles of denaturation at 94°C for 15 seconds, annealing at 58°C for 30 seconds, extension at 68°C for 2 minutes 30 seconds; final extension at 68°C for 5 minutes.

**Direction**	**HCV Genotype**	**Sequence**
**RT-PCR primers**		
Sense	1a	CCCTACTTYGTGCGCGTYCAAGGC
Antisense	1a	TGGTGACRGCAGCTGTAAAAGCCATC
Sense	1b	GACATCATCTYGGGYCTGCCYGTC
Antisense	1b	CGCCTGCTAAGTAYTGTATCCCGCTG
Sense	2a	TTYGTCAGRGCTCACGCTCTG
Antisense	2a	GAGGGCTTCTCGCCAGACATGATYTTRAA
Sense	2b	ATGGCCTCCCGGTYTCCGCGAGGYTAGG
Antisense	2b	GCTTRGGCCATGATGACTGTA
Sense	2c	CCAATGGAAAGGAAGGTCATTGTGTG
Antisense	2c	GAGGGCAGCACTGAAGGACATCAT
Sense	2i	TGGTGATATCATACATGGATTGCC
Antisense	2i	TTGTCTGGACGGCGGGCTGCAC
Sense	2j	ACATTTATGACCACCTCTCCCCTATG
Antisense	2j	CTGGATGCCACTGATGAAATTCCAC
Sense	2k	GTCATTGTGTGGGGAGCGGAG
Antisense	2k	TGCTTCGTGGCTTGTTGTAGC
Sense	2l	GTGGCCTGTGGTGACATCCTAC

Direction	HCV Genotype	Sequence
Antisense	2l	CTGCTTCGAGGCCTGCTGCAGC
Sense	3a	GTCCCATGGAAATCAAGGTCATCACCTG
Antisense	3a	CTCCTCCCATGATCTTAAAAGCCACCAAGG
Sense	3b	GAGATWAAGGTTATCACCTGGGGTGC
Antisense	3b	GCCCTTTGCARCAAGCCGAGGAC
Sense	3i	GCCCCATGGAGATTAAAGTCATTACCTG
Antisense	3i	GTGACCGAGGCAGTGAATGCCAT
Sense	4a, 4g, 4h, 4k, 4o, 4q, 4v	GAAGGTCATMGTCTGGGGCGC
Antisense	4a, 4g, 4h, 4k, 4o, 4q, 4v	GGGCTTGTRACGGCRGCRGTRAAGGACAT
Sense	4c	GAAGGTGATTGTCTGGGGAGC
Antisense	4c	GGGCTTGTGACGGCGGCAGTGAAGGACAT
Sense	4d	AGAAGAAARTCATAACYTGGGGYGC
Antisense	4d	GCCATGAGGGAAGCAATGGTTGG
Sense	4m	GAAAATCATCACGTGGGGTGC
Antisense	4m	GGGCTTGTAACTGCAGCGGTGAACGACAT
Sense	4r	GAAGGTGATTACCTGGGGTGC
Antisense	4r	GGGCTGGTGACGGCAGCAGTGAAGGACAT
Sense	4t	AAAGGTCATAACCTGGGGAGC
Antisense	4t	GGACTTGTGACTGCTGCAGTAAATGACAT
Sense	5a	GCCTTGCTCCACCTTGGTAGGCTG
Antisense	5a	GGGAGGTTACCGCGGCGGTGAAAGACATC
Sense	6a	CCTACTAGCTATCCTGGCCCCTCTATACATCTTGC
Antisense	6a	GCAGGGTCTGAGAAGTCCTCAGAGGACTG
Sense	6b	AGCGACGGCTTRCGHGATCTAGC
Antisense	6b	AAGCCYGCCAGRTACTGAATCC
Sense	6e	CTTCTGGCAATCATTGGCCCCACTTACCTTCTGC
Antisense	6e	GAAGGGTGGTGGAAGTTGAGAGAGGACTGG
Sense	6e (alt)	CGGGTTGGGGCATGGACAGGKACCTAC
Antisense	6e (alt)	CGGGTTGGGGCATGGACAGGKACCTAC
Sense	6f	ATGGAGAAGAARCTGATCACCTGGG
Antisense	6f	TTGCTTGGTCGTGGCCTGGAGCAGCC
Sense	6h	CTTGCTGGCTTTGATTGGTCCGCTCTACCTACTGC
Antisense	6h	GCACCGTACCCGGCTAGGACRTCCACG
Sense	6h (alt)	GAGCCCGTCRTCTTCTCCCCAATGGAG
Antisense	6h (alt)	GCCAAAACTGTTCAAGCTTCGGCC

Direction	HCV Genotype	Sequence
Sense	6i, 6j	GGCCTTCCMGTCTCAGCTAGGCG
Antisense	6i, 6j	AAGGMCATGAGGGAAGCTATTGC
Sense	6l	GTGTTGGCCCTGGCGGGTCCTCTCTACATGC
Antisense	6l	GTTAAGTAGGAGGGTCGTGGAAGTTGACAGTGG
Sense	6m	CACATGGGGCGCTGATACTGTTGC
Antisense	6m	TCGGCCAAGCAGCGTGGACGGCTGGC
Sense	6n	CTGGGGTGCTGACACTGCTGCATGTG
Antisense	6n	CAAGCGGAATGGACAGCCGGTTTAAGC
Sense	6p	GTATGTGCAAGCGTGTCTCTTGAAAATCGG
Antisense	6p	GGAGGGTTGTAGATGTGGCTAAAGGACTTG
Sense	6q	CTGTTRGCCCTCATTGGCCCCTTCTACCTGCTCC
Antisense	6q	GCTCCGTACCCAGCCAAGATGTCCACGATG
Sense	6r	GATACTCCTTGCCATTTTGGGGCCTTTGTACATCTTAC
Antisense	6r	GGAGGGTCGTAGATGTGGACAGAGGACTG
**Nested PCR primers**		
Sense	1a	CCCGTCGGCGCGCCGGGGCCRRGAGATACTGC
Antisense	1a	CATCCCTTGCTCGATGTACGG
Sense	1b	GCCCGTCTCGGCGCGCCGGGGRARGGAGATAC
Antisense	1b	ACCACGGGAGCGGCCGCCTCCGCTTGCTTGGTGGCYGTTTG
Sense	2a	GGAGRTYCTCCTYGGCCCAGCTG
Antisense	2a,	GCTGCCCCTCTTCAATGAGAGCCGC
Sense	2b	GAAGTTCTGCTCGGCCCYGC
Antisense	2b	GCCTGTYTTGTGGCCTGTTG
Sense	2c	GGCTCCCTGTCTCCGCCCGGCTCGG
Antisense	2c	TGAAGTTCCACATGTGCTTGGCCC
Sense	2i	CTGGGCCGGGAGGTCCTGCTG
Antisense	2i	TTGGACTTAAACATCTCGGC
Sense	2j	AGAGCCCATAGTCTTCAGCCCGATG
Antisense	2j	TAGGCCTTGAATCTTAGACTTTAGC
Sense	2k	GTGACATCTTRCACGGGCTGCC
Antisense	2k	GCATTTCCGCTATTCGTTGCC
Sense	2l	TACCGGTCTCCGCTAGGTTGG
Antisense	2l	GCTTTGAACATCTCAGCGACC
Sense	3a	CAGCAGCTTGCGGAGATATTCTTTGCG
Antisense	3a	GGTTGGTCGTCAGGGGACTGGTGACTG
Sense	3b	GACATCYTGTGCGGGTTGCCCGTTTC

Direction	HCV Genotype	Sequence
Antisense	3b	GCGATRGCCTGTGCYTGCTCGATG
Sense	3i	GCGGGTTGCCTGTCTCGGCGCGC
Antisense	3i	GACAAAGTTCCACATATGTTTGCGCC
Sense	4a, 4g, 4h, 4k, 4o, 4q, 4v	GGCAATGAGRATHTTGCTCGGCCC
Antisense	4a, 4g, 4h, 4k, 4o, 4q, 4v	TTCCACATGTGYTTYGCCCARAAYTG
Sense	4c	GGCAATGAGATCCTGCTTGGGCC
Antisense	4c	TTCCACATGTGCTTGGCCCAGAACTG
Sense	4d	TGGGYAATGAAATCTTGCTCGGC
Antisense	4d	GAAGTTCCACATATGCTTCGCC
Sense	4m	GGCAATGAAATCTTGCTCGGTCC
Antisense	4m	TTCCACATGTGTTTCGCCCARAACTG
Sense	4r	GGCAATGAAATATTGCTTGGCCC
Antisense	4r	TTCCACATGTGTTTTGCCCAGAATTG
Sense	4t	GGCAATGAGATCTTGCTTGGGCC
Antisense	4t	TTCCACATATGCTTAGCCCAAAACTG
Sense	5a	CCCATGAAGGATTGGGCAGCGTCCG
Antisense	5a	GCCAAGTATTGGATCCCACTGACAAAGTTCCAC
Sense	6a	GCGACGGACTGCGCGATCTGG
Antisense	6a	GCCAYGTACTGAATGCCGCTGACAAAGTTCCAC
Sense	6b	GGACCCGCRGATGACATGCGTC
Antisense	6b	CCACGTGGARTGRATAGCGGG
Sense	6e	CGTGCAGGCCGCCCTGTTACGGG
Antisense	6e	GCGAGATACTGTATCCCGCTAACGAAATTCC
Sense	6e (alt)	GTGTTCTCGCCTATGGAGAAGAA
Antisense	6e (alt)	TTTTGTTTGAACTGCTCAGCAAG
Sense	6f	CTTGYGGTGACATTATAGCTGGCC
Antisense	6f	CTGTTCAGCTATTTGTTGACCTTCG
Sense	6h	CGGCTTGCGAGATCTAGCTGTCGC
Antisense	6h	CAGCTAGGTACTGGATGCCACTCACGAAATTCC
Sense	6h (alt)	CTGCCTGCGGCGAYATYATCGCCGGTC
Antisense	6h (alt)	GTTTGCTTGTGGATTGTATGAGGCC
Sense	6i, 6j	TGGGSCCCGCTGATGACGTC
Antisense	6i,6j	ATGGCTTGRGCCTCAGGGAGG
Sense	6l	GGCGWGCAACGGCCTACGAGATC
Antisense	6l	CAAGATATTGAATGCCGCTCACAAAGTTCC

Direction	HCV Genotype	Sequence
Sense	6m	CAACCTCGTCTTTCTTGGTCCTGC
Antisense	6m	TGAATTGTTCTGCAATAGCCCGGCC
Sense	6n	GCACGGAAGGGTAATCTTATCCTTCTTG
Antisense	6n	GTTTGAACTGCTCTGCTATGAGCTGGC
Sense	6p	GCGACGGATTGAGAGATCTTGCCG
Antisense	6p	GCCAGGTATTGAATCCCACTGACAAAGTTCC
Sense	6q	CAGCGACGGCTTACGAGACCTTGC
Antisense	6q	CCGGCTAGGTACTGTATCCCACTGATGAAGTTCC
Sense	6r	GAGCAACGGCCTGCGTGACCTTGC
Antisense	6r	CCAGATACTGTATCCCGCTGATGAGGTTCC

Supplemental Table S5. Amplification of NS5A from HCV-infected clinical samples

RT-PCR reaction was conducted using SuperScript III One-Step RT-PCR System with Platinum Taq High Fidelity (Invitrogen) using cycling conditions: cDNA synthesis at 55°C for 30 minutes; denaturation at 94°C for 2 minutes; PCR amplification with 40 cycles of denaturation at 94°C for 2 minutes, annealing at 55°C to 58°C for 30 seconds, extension at 68°C for 2 minutes to 2 minutes 30 seconds; final extension at 68°C for 5 minutes.

Nested PCR reaction was conducted using Platinum Pfx DNA Polymerase (Invitrogen) using cycling conditions: denaturation at 94°C for 5 minutes; PCR amplification with 35 cycles of denaturation at 94°C for 15 seconds, annealing at 55°C to 58°C for 30 seconds, extension at 68°C for 2 minutes to 2 minutes 30 seconds; final extension at 68°C for 5 minutes.

Direction	HCV Genotype	Sequence
**RT-PCR primers**		
Sense	1a	GCAATGGATGAACCGGYTRATAGCCTTCGCCTC
Antisense	1a	CTCAGGCTGAACRCAGAAAACC
Sense	1b	GCGTTCGCTTCGCGGGGTAACC
Antisense	1b	CTCTGGTTGGACRCAGAAAACC
Sense	2a	GATCATGTCTGGCGAGAAGCCCTC
Antisense	2a	ACGCAGAACACCTCATTTTTGGCC
Sense	2b	TAGCTTTTAARATCATGAGCGGCG
Antisense	2b	TGTTGGTCTTCCAGGAGGTCCTCC
Sense	2c	TTYAAGATCATGTCTGGCGAGAARCCCWC
Antisense	2c	GAACACCTCGTTYTTGGCCATGATGGTTGT
Sense	2i	GGAAGGYGCGGTCCAATGGATGAAYAGGCT
Antisense	2i	TCCARGAGGTCCTTCCACACGGACTTGATG
Sense	2l	AAAAGCCCACCATGGAGGATGTGGTCAACA
Antisense	2l	GTCCACGCAGAACACCTCATTCTTTGCCAT
Sense	3a	GGRGCGGTVCAGTGGATGAACAG
Antisense	3a	GTCTTRTARTGGTCGTCGAGCAC
Sense	3b	GRGAAGGAGCAGTGCAGTGGATGAACAGACT

Direction	HCV Genotype	Sequence
Antisense	3b	TCCARCAAGTCCTCCCAGACGGARCGGATC
Sense	4a, 4f, 4g, 4o, 4m, 4p, 4 – other subtypes	ARGGGGCYGTGCAGTGGATGAACCG
Antisense	4a, 4c	ARGCGGTCRAAGGTYACCTTYTTCTG
Sense	4d	AGGGRGCRGTCCAGTGGATGAACCG
Antisense	4d, 4f	ATGCGGTCGAAGGTGACYTTCTTYTG
Sense	4c	ARGGGGCYGTGCAGTGGATGAATCG
Sense	4b	AGGGGGCYGTRCAGTGGATGAAYCG
Antisense	4b	AGGCGGTCRAAWGTGACYTTYTTCTG
Antisense	4g	AKRCGGTCRAARGTGACCTTCTTCTG
Antisense	4o, 4m, 4p, 4 – other subtypes	CGGTCRAAGGTSACCTTCTTYTGCCG
Sense	5a	CCTCCTGCCYTCAATACTCTGCCCTGGAGC
Antisense	5a	GAGGARGTGGARTACACAAGATTGTGGTGGCG
Sense	6a, 6b	GTGTGTGCTGCCATCTTAAGACGCCACGTTGG
Antisense	6a, 6b	TCTGTCAAATGTGACCTTCTTCTGGCGGAG
Sense	6e, 6u	TCATGAGCGGCGAGGCCCCTGCTTTAGAGGACA
Antisense	6e, 6u	GTCTTCCAGCAAGTCCTCCCACACGGAGTTGATGT
Sense	6f, 6r	GACGGCCTTTGTCGTTAGCGGCATGGCYGGC
Antisense	6f, 6r	GTCCTCCCACACGGAGTTGATGTGGTTAATGGCC
Sense	6h, 6m	GCTTGGCCGAAGCTTGAACAGTTTTGGCAAAA
Antisense	6h, 6m	CCCACACGGAGTTGATGTGGTTAATGGCCTT
Sense	6p, 6q, 6t	YCAGTGGATGAACAGGCTCATAGCGTTYGC
Antisense	6p, 6q, 6t	CACACGGAGTTGATGTGGTTAATGGCYTTGCT
Sense	6l	GTGTGTGCGGGCATYCTRAGGCGTCACGC
Antisense	6l	ATCGYTGTAGTGTTGGTCTAGCAGTTGCACACG
**Nested PCR primers**		
Sense	1a	GCCGGAGAGCGATGCGGCCGCCCGCGTCACTGCCATACTCAGC
Antisense	1a	CCACACGGAGTTGATGTGG
Antisense	1a	CTCCAGGGGAGGCATCGATGAATAGGACTCAACGTCGGAGTCG
Sense	1b	GCCTGAGAGCGACGCGGCCGCGCGTGTCACYCAGATCCTYTC
Antisense	1b	CCTTCATCTCCTTRAGCACGTCCYGGTARTG
Antisense	1b	CTCAAGGGGAGGCATCGATGAGTACGACTCAACGTCGGATCC
Sense	2a	CCACGTCGCCCCYACCCACTACGTGACGGAR
Antisense	2a	TCCTTCCACACGGACTTGATGTGG
Antisense	2a	CCTGGCCAGCCTACGCTTAGCAGCCTCCGCCGTGATATGGGACG
Sense	2b	GGGCGGTCCAGTGGATGAACAG

Direction	HCV Genotype	Sequence
Antisense	2b	CAGGAGGTCCTCCCACACGGACCG
Antisense	2b	CCTGGCCAGCCTACGCTTAGCCGCCTCCGCTGTAATGTGGGACG
Sense	2c	AAGGCGCGGTCCAATGGATGAATAGGCTCA
Antisense	2c	GGTCTTCCAGGAGGTCCTCCCACACGGACT
Sense	2i	GTCCAGAGGTAACCAYGTCGCCCCRACRCA
Antisense	2i	CCRAATCCGTACTTGGATCGTGCAGAATG
Sense	2l	GGAAGGTGCGGTCCAATGGATGAACAGGCT
Antisense	2l	CTTGATGTGGTTAACGGCCCKCCTGGATAA
Sense	3a	CGAGAGCGATGCTGCAGCRARGGTCAC
Antisense	3a	CTGACGYTGAGAAGCGCTTCTAGACGACG
Antisense	3a	CCTGGCCAGCCTACGCTTAGCCGTCTCGGCGGTGATATGGGAAG
Sense	3b	TCACCAACACAYTATGTCCCYGAGAGCGACG
Antisense	3b	GTCATCGAGCACYTGCAGYCTGTCGAAGGTA
Sense	4a, 4f, 4g, 4o, 4m, 4p, 4 – other subtypes	GGCAATCACGTGTCTCCYACGCAYTAYGTC
Antisense	4a, 4c	GCTRATTGGCAGCTTYGATTCYTCAGC
Sense	4d	GYAACCACGTGGCTCCCACRCACTACGT
Antisense	4d	GTTRATGGGCAGCTTGGTYTCYTCAGC
Antisense	4f	TTRATRGGCAGCTTGGACTCYTCMGCT
Sense	4c	GGCAATCAYGTGKCGCCCACGCACTA
Antisense	4g	ATKGGYAATTTGGYCTCYTCRGCCGC
Sense	4b	GGCAATCAYGTGTCSCCYACRCACTAYGT
Antisense	4b	ATTGGYAGTTTYGGCTCTTCRGCCG
Antisense	4o, 4m, 4p, 4 – other subtypes	GGGCTRATTGGCAGCTTKGWTTCYTC
Sense	5a	CAGTGGATGAATAGGCTGATYGCGTTCGC
Antisense	5a	TTCCTCCTCAGCRGAGCARGGGGTGATGAG
Sense	6a, 6b	AACCAGTGGATGAACAGGCTAATAGCCTTTGC
Antisense	6a, 6b	CTTCTCCTCCTCCGCAGCRCATGGTGTTAT
Sense	6e, 6u	CCCCYACGCACTACGTCCCAGAGACAGAYGC
Antisense	6e, 6u	CCCCGTACCCGAACTTGGAGCGAGCCGAGTG
Sense	6f, 6r	AATCAGTGGATGAACMGACTGATAGCCTTYGCCTC
Antisense	6f, 6r	CYTCCTCCGTGGAGAGGAGCCTRGCGCTCA
Sense	6h, 6m	CCAGTGGATGAACAGGCTAATMGCYTTTGCCTCT
Antisense	6h, 6m	CCTTCACCTGGGACGCCCGGAGCTTARTCTC
Sense	6p, 6q, 6t	ACRCACTAYGTGCCYGAGACGGACGCCTC
Antisense	6p, 6q, 6t	CAGRTTGTGGTGGCGTATGAGRGAGTTRCTCA
Sense	6l	CAGTGGATGAACCGRCTRATAGCGTTCGCYTC

Direction	HCV Genotype	Sequence
Antisense	6l	CTCCGAGATGTCGTGGAGTACACCAGRTTATG
